# m^5^C-dependent cross-regulation between nuclear reader ALYREF and writer NSUN2 promotes urothelial bladder cancer malignancy through facilitating RABL6/TK1 mRNAs splicing and stabilization

**DOI:** 10.1038/s41419-023-05661-y

**Published:** 2023-02-18

**Authors:** Ning Wang, Ri-xin Chen, Min-hua Deng, Wen-su Wei, Zhao-hui Zhou, Kang Ning, Yong-hong Li, Xiang-dong Li, Yun-lin Ye, Jun-hua Wen, Biao Dong, Xue-pei Zhang, Zhuo-wei Liu, Fang-jian Zhou

**Affiliations:** 1 State Key Laboratory of Oncology in South China, Collaborative Innovation Center for Cancer Medicine, Sun Yat-sen University Cancer Center, Guangzhou, 510075 China; 2 Department of Urology, Sun Yat-sen University Cancer Center, Guangzhou, 510075 China; 3 Department of Urology, The First Affiliated Hospital of Zhengzhou University, Zhengzhou, 450003 China; 4 Department of Thoracic Surgery, Guangdong Provincial People’s Hospital (Guangdong Academy of Medical Sciences), Southern Medical University, Guangzhou, 510080 China; 5 Cancer Prevention Center, State Key Laboratory of Oncology in South China, Guangzhou, 510075 China

**Keywords:** Bladder cancer, Cancer epidemiology

## Abstract

The significance of 5-methylcytosine (m^5^C) methylation in human malignancies has become an increasing focus of investigation. Here, we show that m^5^C regulators including writers, readers and erasers, are predominantly upregulated in urothelial carcinoma of the bladder (UCB) derived from Sun Yat-sen University Cancer Center and The Cancer Genome Atlas cohort. In addition, NOP2/Sun RNA methyltransferase family member 2 (NSUN2) as a methyltransferase and Aly/REF export factor (ALYREF) as a nuclear m^5^C reader, are frequently coexpressed in UCB. By applying patient-derived organoids model and orthotopic xenograft mice model, we demonstrate that ALYREF enhances proliferation and invasion of UCB cells in an m^5^C-dependent manner. Integration of tanscriptome-wide RNA bisulphite sequencing (BisSeq), RNA-sequencing (RNA-seq) and RNA Immunoprecipitation (RIP)-seq analysis revealed that ALYREF specifically binds to hypermethylated m^5^C site in *RAB, member RAS oncogene family like 6 (RABL6)* and *thymidine kinase 1 (TK1)* mRNA via its K171 domain. ALYREF controls UCB malignancies through promoting hypermethylated *RABL6* and *TK1* mRNA for splicing and stabilization. Moreover, ALYREF recognizes hypermethylated m^5^C site of *NSUN2*, resulting in NSUN2 upregulation in UCB. Clinically, the patients with high coexpression of ALYREF/RABL6/TK1 axis had the poorest overall survival. Our study unveils an m^5^C dependent cross-regulation between nuclear reader ALYREF and m^5^C writer NSUN2 in activation of hypermethylated m^5^C oncogenic RNA through promoting splicing and maintaining stabilization, consequently leading to tumor progression, which provides profound insights into therapeutic strategy for UCB.

## Introduction

RNA epigenetic modifications, including N^6^-methyladenosine (m^6^A) [1], have been widely implicated functioning in various cellular, developmental, and pathological processes, and determine the fate of RNAs [2, 3]. As one of the most common RNA modification, 5-methylcytosine (m^5^C) has been identified in tRNAs, rRNAs and mRNAs [4–7] and plays an essential role in RNA metabolism [8]. The recent research [9] showed that metastasis-initiating tumor cells require mitochondrial m^5^C to activate invasion and dissemination. mRNA m^5^C methylation was initially catalyzed by NOP2/Sun RNA methyltransferase family member 2 (NSUN2) and enriched in the vicinity of translational start codon and 3′ untranslated region (UTR) [10, 11]. Huang et al. [12] reported an improved method to identify mRNA m^5^C sites and determined sequence motifs. Li et al. [13] stratified m^5^C sites to two types: type I m^5^C sites contained a downstream G-rich triplet motif; type II m^5^C sites contain a downstream UCCA motif. Aly/REF export factor (ALYREF) has been identified as the first nuclear m^5^C reader [14]. Y-box protein 1 (YBX1) [15, 16] has been characterized as the first cytoplasmic m^5^C reader, maintaining the stability of its targeted m^5^C transcripts.

So far, it has been evidenced that m^6^A RNA methylation played an important role in cancer occurrence and development [1, 17–19]. The significance of m^5^C methylation in human malignancies has become an increasing focus of investigation. It was reported that activation of RNA m^5^C modification was critical for tumor-initiating cells fate and global protein synthesis [20]. We have previously revealed that m^5^C is preferentially hypermethylated in urothelial carcinoma of the bladder (UCB) and represents a novel mechanism for oncogene activation [21]. As the governor of m^5^C methylation, m^5^C regulators, including writers, readers and erasers, play central roles in tumor pathogenesis. Dysregulated expressed m^5^C regulators in human cancers have been reported in several studies [22–24]. We previously reported that cytoplasmic m^5^C reader YBX1 stabilizes *HDGF* mRNA, leading to enhanced tumor progression in UCB [21]. m^5^C writer NSUN2 acts as an oncogene to promote gastric cancer [25] and hepatocellular carcinoma (HCC) [26] development by regulating protein-encoding gene CDKN1C and lncRNA H19, respectively. Wang et al. [27] reported that m^5^C-methylated *PKM2* mRNA was recognized by ALYREF and promoted the glucose metabolism in UCB. Despite these studies, more detailed investigations focusing on the collaboration network among these m^5^C regulators are still lacking.

UCB is one of the most malignant cancers [28], with a recurrence rate of up to 74% among non-muscle-invasive bladder cancer patients [29]. For muscle-invasive bladder cancer, up to 50% of patients die from distant metastases despite undergoing radical cystectomy with pelvic lymph node dissection [30]. It was reported that 70%-80% bladder cancer patients occurred mutations in the promoter of the gene encoding telomerase reverse transcriptase [31]. Deletions in chromosome 9 [32], mutations in FGFR3 [33] and PI3K [34] were seen as early oncogenic events in UCB. In addition, epigenetic dysregulation may also contribute to the progression of bladder cancer [35]. Our previous studies unveil a novel regulatory mechanism of oncogene activation mediated by m^5^C methylation in UCB. It is critical to further identify genome-wide m^5^C methylated genes that function in UCB tumorigenesis.

In this study, we demonstrate that m^5^C regulators are predominantly upregulated in UCBs from Sun Yat-sen University Cancer Center (SYSUCC) and The Cancer Genome Atlas (TCGA) cohort, and m^5^C writer NSUN2 and nuclear m^5^C reader ALYREF are frequently coexpressed. Patient-derived organoids model and orthotopic xenograft mice model showed that ALYREF promotes proliferation and invasion of UCB cells in an m^5^C-dependent manner. Integration of transcriptome-wide RNA-bisulphite sequencing (BisSeq), RNA-sequencing (RNA-seq) and RNA Immunoprecipitation (RIP)-seq analysis revealed that ALYREF specifically binds to hypermethylated m^5^C site in *RAB, member RAS oncogene family like 6 (RABL6)* and *thymidine kinase 1 (TK1)* mRNA via its K171 domain. Mechanistically, ALYREF controls UCB malignancies through promoting hypermethylated *RABL6* and *TK1* mRNA for splicing and maintaining stabilization. Moreover, ALYREF recognizes hypermethylated m^5^C site of *NSUN2*, resulting in NSUN2 upregulation in UCB. Clinically, triple expression of high levels of ALYREF/RABL6/TK1 predict the poorest survival.

## Results

### m^5^C regulators are predominantly upregulated in UCB

**Fig. 1:**
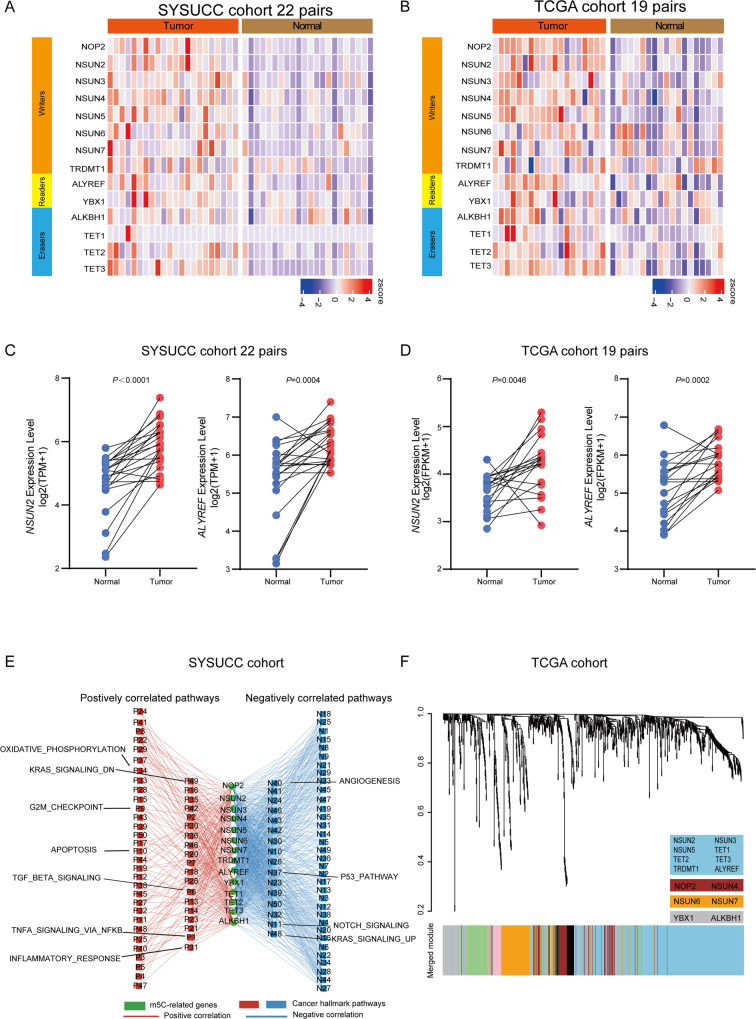
m^5^C regulators are predominantly upregulated in UCB. **A**, **B** Heat maps of m^5^C regulator mRNA expression profiles in paired UCB tumor tissues and adjacent normal tissues (22 pairs from SYSUCC cohort [**A**] and 19 pairs from TCGA cohort [**B**]). **C**, **D** The expression levels of ALYREF and NSUN2 (fold change > 1.1, *P* <0.05) in the SYSUCC cohort (22 pairs) (**C**) and TCGA cohort (19 pairs) (**D**). Data represent the mean ± S.D., and a two-tailed paired Student’s *t*-test was applied to determine the *P-*value. **E** The signal pathways in which m^5^C regulators may be involved from RNA-seq on SYSUCC UCB cohort. Positive correlations are shown in red; negative correlations are shown in blue. **F** WGCNA showing the networks among m^5^C regulators in the hierarchical cluster tree.

To investigate the roles of m^5^C regulators in UCB malignancy, we analyzed the expression profile of m^5^C regulators in UCB derived from SYSUCC and TCGA cohort. By analyzing our previously published RNA-seq data of 22 paired normal and adjacent UCB tumor tissues, we identified that 7 writers (NOP2, NSUN2, NSUN3, NSUN4, NSUN5, NSUN6 and NSUN7), one reader (ALYREF) and two erasers (TET2 and TET3) were statistically significant and recurrent upregulated in UCB tumor tissues (fold change > 1.1, *P*-value <0.05, occurrence rate> 50%) (Fig. 1A). Moreover, five writers (NOP2, NSUN2, NSUN3, NSUN4 and NSUN5), one reader (ALYREF) and two erasers (TET2 and TET3) were consistently upregulated in UCB tumor tissues compared to adjacent normal tissues from TCGA cohort (fold change > 1.1, *P*-value <0.05, occurrence rate > 50%) (Fig. 1B). Among these two cohorts, we found that the expression of ALYREF and NSUN2 were significantly upregulated in UCB tumor tissues compared to adjacent normal tissues (Fig. 1C, D). The expression pattern of other m^5^C regulators were shown in Fig. S1A and S1B.

To investigate the potential function of these m^5^C regulators in UCB, we analyzed the signal pathways in which m^5^C regulators may be involved from RNA-seq on SYSUCC UCB cohort. The expression levels of m^5^C regulators were positively associated with multiple oncogenic pathways, such as K-RAS signaling, oxidative phosphorylation, and TGF-β signaling. Meanwhile, tumor suppressor pathway, such as P53 pathway was negatively associated with m^5^C regulator expression (Fig. 1E). These results together suggest the essential role of m^5^C regulators in cancer progression.

To explore the networks among m^5^C regulators, we conducted Weighted Gene Coexpression Network Analysis (WGCNA) on the UCB TCGA cohort. Notably, m^5^C writers (NSUN2, NSUN3 and NSUN5) and erasers (TET1, TET2 and TET3) were coexpressed with nuclear reader ALYREF in an mRNA module (Fig. 1F). Our finding indicates that cross-talks among writers, erasers and readers may exist in the m^5^C regulation, in which ALYREF serves as a core factor, and some m^5^C regulators may function synergistically. Collectively, these results strongly support that m^5^C regulators may link to UCB pathogenesis.

Next, we explored the potential function of these m^5^C regulators candidates in UCB cells. NSUN3, NSUN5, TET2 and TET3 were knocked down by siRNAs in T24 cells (Fig. S1C). Colony-formation and migration assays demonstrated that these four m^5^C regulators have no significant roles in the proliferation and migration of UCB cells (Fig. S1D and E).

### ALYREF is upregulated in UCB and correlates with poor overall survival (OS) in UCB patients

**Fig. 2:**
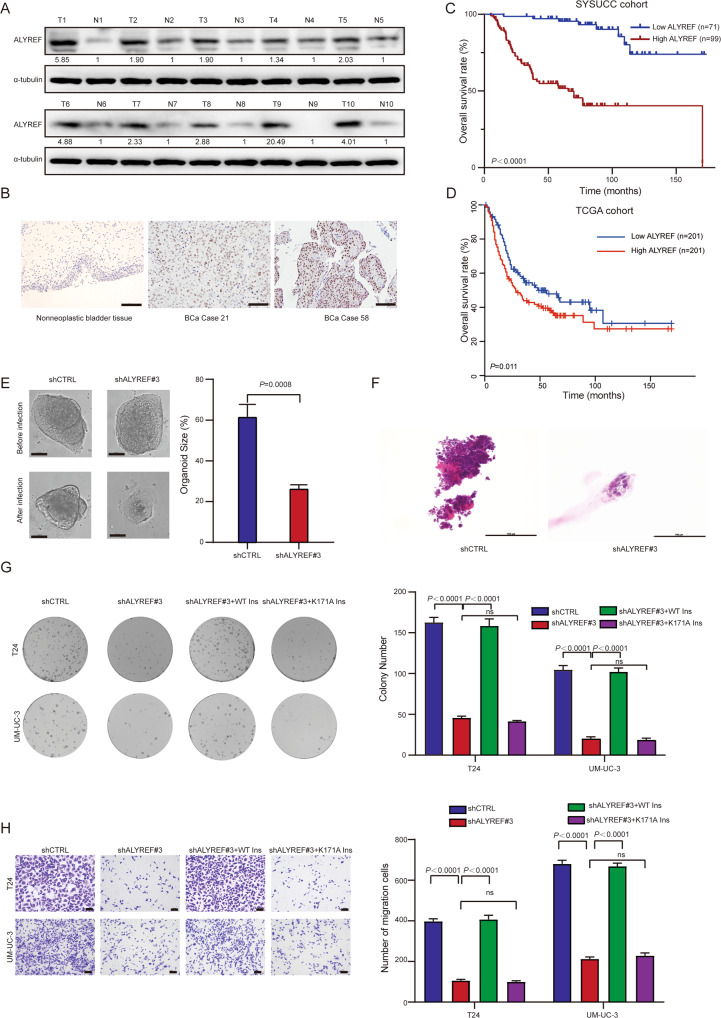
ALYREF is upregulated in UCB and enhances UCB cell proliferation and invasion as an m^5^C reader in vitro. **A** Western blotting showing ALYREF expression in 10 pairs of UCB and adjacent non-neoplastic tissues. The expression was normalized by α-tubulin expression. T, tumor tissue; N, nonneoplastic bladder tissues. **B** IHC staining assays and representative images of ALYREF in nonneoplastic bladder tissues (Left) and UCB tissues (Medium and Right). Scale bars, 100 μm. **C** Kaplan–Meier analysis showing that upregulated ALYREF predicts poor OS in the SYSUCC cohort. The *P*-value was calculated by a log-rank test. **D** Kaplan–Meier analysis showed patients with high mRNA expression level of *ALYREF* predicted poorer OS. The *P*-value was calculated by a log-rank test. The group cutoff was 50%, which was the expression threshold for splitting the high-expression and low-expression cohorts. The data were from the public database http://gepia2.cancer-pku.cn/#index. **E** Organoid model showing the growth effect transfected with shCTRL and shALYREF#3 (Left). Statistical analysis of organoid size after 7 days of infection with shCTRL and shALYREF#3 (Right). Scale bars, 100 μm. Data represent the mean ± S.D., *n* = 3, and a two-tailed unpaired Student’s *t*-test was applied to determine the *P-*value. **F** Representative Hematoxylin-eosin staining images of organoids after infection with shCTRL (Left) and shALYREF#3 (Right). Scale bars: 100 μm. **G** Colony forming assay showing the effect of ALYREF with a WT m^5^C site on the restoration of cell growth in ALYREF-knockdown cells relative to ALYREF with K171A mutant. Left: representative images of cell colonies in T24 (Top) and UM-UC-3 (Bottom) cells; Right: histograms of colony numbers. Data represent the mean ± S.D., *n* = 3. A two-tailed unpaired Student’s *t*-test was applied to calculate the *P*-value. **H** Migration assay showing the effect of ALYREF with a WT m^5^C site on the restoration of cell migration in ALYREF-knockdown cells relative to ALYREF with K171A mutant. Left: representative images of migration cells in T24 (Top) and UM-UC-3 (Bottom) cells; Scale bars, 100 μm. Right: histograms of the number of migration cells. Data represent the mean ± S.D., *n* = 3. A two-tailed unpaired Student’s *t-*test was applied to calculate the *P-*value.

We investigated the clinical significance of ALYREF expression in UCB. Western blotting assay of samples from 10 UCB patients from SYSUCC showed that ALYREF was frequently upregulated in UCB tissues (Fig. 2A). Immunohistochemistry (IHC) staining was performed in a cohort of 170 UCB tissues and 30 paired nonneoplastic bladder tissues from SYSUCC. High ALYREF expression was observed in 99/170 (58.2%) UCB patients. The representative IHC staining images in nonneoplastic bladder tissues and UCB tissues were shown in Fig. 2B. High expression of ALYREF was associated with poor OS in UCB patients significantly (Fig. 2C). The http://gepia2.cancer-pku.cn/#index., which analyzed TCGA cohort, showed that patients with high mRNA level of *ALYREF* predicted poorer OS (Fig. 2D). These data provide evidence that ALYREF is a potential oncogene in human UCB.

### ALYREF enhances UCB cell proliferation and invasion in an m^5^C-dependent manner

Patient-derived organoids serve as an ideal cell model to study tumor pathogenesis [36–38]. To further explore the role of ALYREF in UCB aggressiveness, we constructed a patient-derived organoid in vitro. ALYREF was knocked down in T24 and UM-UC-3 cells by two short hairpin RNAs (shRNA-2 and shRNA-3) (Fig. S2A). We found that UCB organoid growth was significantly inhibited after knockdown of ALYREF (Fig. 2E, F). Colony-formation and migration assays demonstrated that cell growth and migration abilities were largely reduced after knockdown of ALYREF (Fig. S2B and S2C). We further found that ALYREF did not affect cell growth and migration abilities in a normal urothelial cell line, SV-HUC-1 (Fig. S2A, S2D and S2E). We then explored if the oncogenic function of ALYREF relies on m^5^C recognition capacity. It has been reported [14] that ALYREF K171A mutation led to a strongly reduced ALYREF binding ability to m^5^C-containing oligonucleotide, we therefore examined whether ALYREFY K171A mutation could affect the function of ALYREF. We overexpressed shALYREF#3-insensitive wild-type (WT) or the K171A-mutant ALYREF in ALYREF-depleted UCB cells, respectively. The downregulated expression of ALYREF in ALYREF-depleted UCB cells was rescued by overexpression WT or the K171A-mutant ALYREF (Fig. S2F). We further found that after knockdown of ALYREF, colony-formation and cell counting kit-8 (CCK8) assays showed that the reduced tumor cell growth could be rescued by the overexpression of WT ALYREF, but not the K171A-mutant ALYREF (Fig. 2G and Fig. S2G). Migration and invasion assays showed that the reduced migration and invasion capacity could be rescued by the overexpression of WT ALYREF, but not the K171A-mutant ALYREF (Fig. 2H and Fig. S2H). On the contrary, the cell growth and migration abilities of the cells subjected to ALYREF overexpression were significantly increased compared with those of control cells (Fig. S2A, S2I and S2J). Our data indicate that ALYREF exerts oncogenic effects in UCB cells in an m^5^C-dependent manner.

**Fig. 3:**
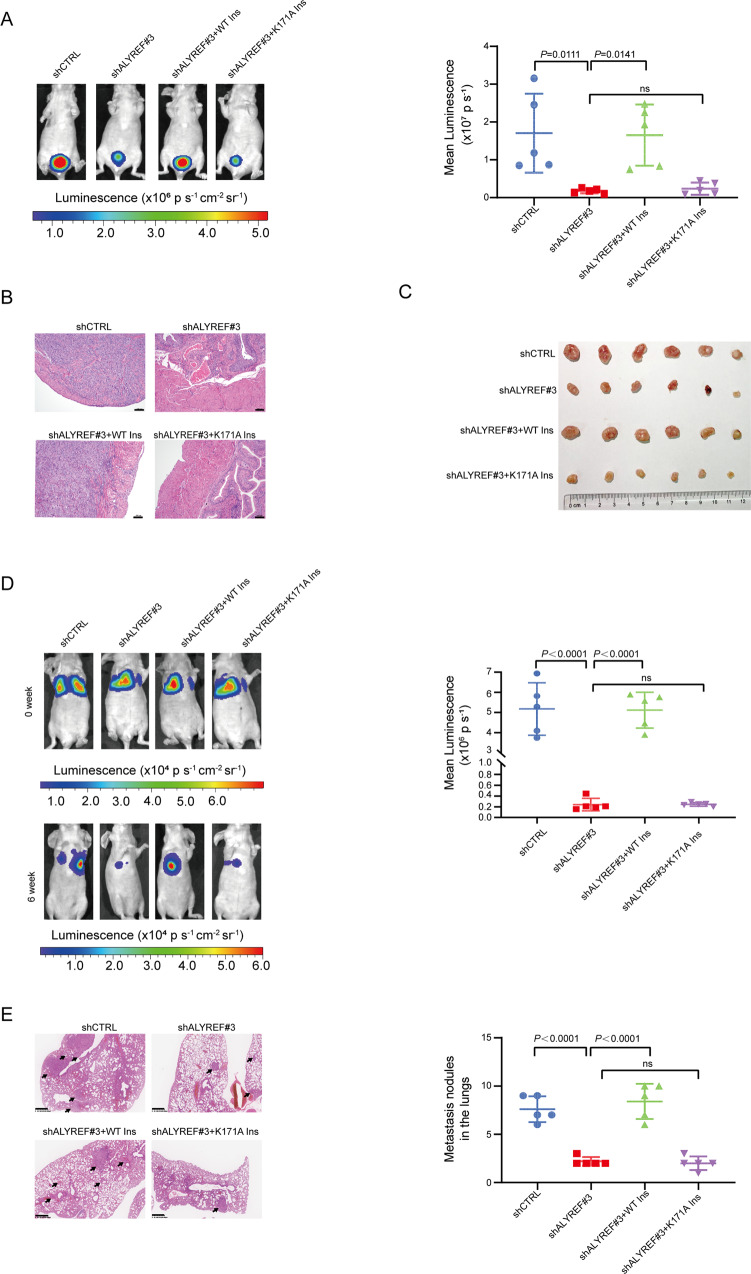
ALYREF promotes UCB pathogenesis and metastasis as an m^5^C reader in vivo. **A** The orthotopic xenograft model showing the effect of ALYREF with a WT m^5^C site on the restoration of submucosal lesions in ALYREF-knockdown cells relative to ALYREF with K171A mutant. Left: representative bioluminescence images; Right: statistical results for the bioluminescence signals. Data show the mean ± S.D. The *P-*values were calculated by a two-tailed unpaired Student’s *t*-test. *n* = 5, ns: no significance. **B** Representative Hematoxylin-eosin staining images in different groups of orthotopic xenograft models. Scale bars, 100 μm. **C** The subcutaneous xenograft model showing the effect of ALYREF with a WT m^5^C site on the restoration of subcutaneous tumor formation in ALYREF-knockdown cells relative to ALYREF with K171A mutant. **D** The lung metastasis model showing the effect of ALYREF with a WT m^5^C site on the restoration of tumor metastasis in ALYREF-knockdown cells relative to ALYREF with K171A mutant. Left: representative bioluminescence images are shown at 0 and the 6th week after injection; Right: statistical results for the mean bioluminescence signals in different groups at the 6th week. Data show the mean ± S.D. The *P-*values were calculated by a two-tailed unpaired Student’s *t*-test. *n* = 5, ns: no significance. **E** Left: Hematoxylin-eosin staining and metastatic nodules (indicated by arrows) in lung tissues from different groups at the 6th week. Scale bars: 400 µm; Right: Statistical results for the number of metastatic nodules in the lung among different groups at the 6th week. Data show the mean ± S.D, The *P-*values were calculated by a two-tailed unpaired Student’s *t*-test. *n* = 5, ns: no significance.

An orthotopic xenograft model was used to investigate the role of ALYREF in UCB aggressiveness in vivo. Depletion of ALYREF caused fewer submucosal lesions in mice bladder, and this effect could be rescued by overexpression of WT ALYREF but not K171A-mutant ALYREF (Fig. 3A, B and Fig. S3A and S3B). Tumorigenicity assays in vivo showed that knockdown of ALYREF inhibited subcutaneous tumor formation abilities. However, the reduction of subcutaneous tumor formation abilities could be rescued by the overexpression of WT ALYREF but not the K171A-mutant of ALYREF (Fig. 3C and Fig. S3C). Tail-vein injection metastasis assays in vivo demonstrated that ALYREF depletion inhibited lung metastatic nodules formation. The effect of ALYREF knockdown on tumor cell invasion and lung metastasis was rescued by overexpression of WT ALYREF rather than the mutant (Fig. 3D, E). Together, these results show that ALYREF promotes UCB cell proliferation and invasion in an m^5^C-dependent manner.

### ALYREF targets hypermethylated m^5^C site in RABL6 and TK1 mRNA

**Fig. 4:**
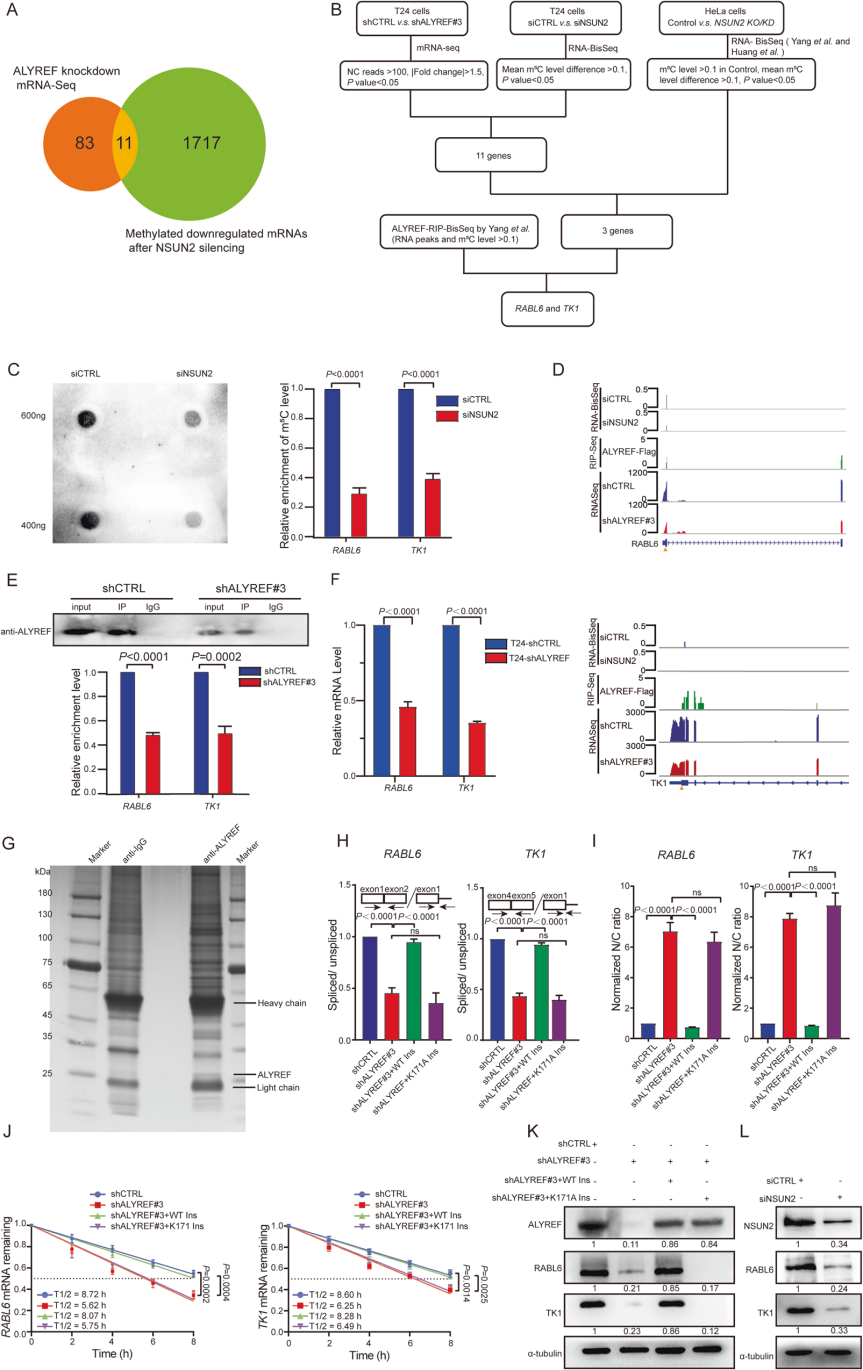
ALYREF facilitates *RABL6* and *TK1* mRNA splicing and maintains mRNA stabilization via targeted the hypermethylated m^5^C mRNA. **A** Venn diagram showing downregulated mRNAs after ALYREF was knocked down and low methylated mRNAs after NSUN2 silencing in T24 cells. Eleven mRNAs are in the intersection. **B** A flowchart illustrated the screening strategy of ALYREF/NSUN2 targeted candidate genes through m^5^C regulation. **C** Silencing NSUN2 reduced the enrichment of m^5^C level in *RABL6* and *TK1*. Left: Dot blotting of m^5^C in siCTRL and siNSUN2 in T24 cells. Right: m^5^C-RIP-qRT-PCR showing the m^5^C level of *RABL6* and *TK1* in siCTRL and siNSUN2 cells. Data represent the mean ± S.D., *n* = 3. A two-tailed unpaired Student’s *t-*test was applied to calculate the *P-*value. **D** Integrative- genomics-viewer tracks representing the read regions of *RABL6* (Top) and *TK1* (Bottom) in shALYREF#3 RNA-seq data, the m^5^C sites when NSUN2 was silenced and the ALYREF-binding regions in the RIP-seq data. The triangle indicates the m^5^C site in *RABL6* (chr9: 139702478) and in *TK1* (chr17: 76170268), respectively. **E** RIP assays showing the association of ALYREF with the m^5^C sites of *RABL6*, and *TK1* mRNAs. Upper panel: western blotting shows the ALYREF IP efficiency in control and shALYREF#3 cells. Bottom panel: Relative enrichment representing *RABL6*, and *TK1* mRNA levels associated with ALYREF compared to an input control. IgG antibody used as a control. Data show the mean ± S.D., *n* = 3. The *P*-values were calculated by a two-tailed unpaired Student’s *t*-test. **F** qRT-PCR showing the expression change of *RABL6* and *TK1* mRNA after ALYREF knockdown. (|log2FC | = 1.125, *P* <0.0001 for *RABL6* and |log2FC | = 1.503, *P* <0.0001 for *TK1*). Data show the mean ± S.D., *n* = 3. The *P*-values were calculated by a two-tailed unpaired Student’s *t*-test. **G** Silver staining assays showing the protein bands binding to endogenous ALYREF. **H** The splicing efficiency of *RABL6* (Left) and *TK1* (Right) mRNA were calculated by the ratio of spliced to unspliced transcripts. Schematic illustration showing the qRT-PCR primers designed across exon-intron junction and across exon-exon junction. Data show the mean ± S.D., *n* = 3. The *P*-values were calculated by a two-tailed unpaired Student’s *t*-test. **I** Cytoplasmic and nuclear mRNA fractionation experiment showing the effect of ALYREF with a WT m^5^C site on the restoration of increased nuclear *RABL6* and *TK1* content in ALYREF-knockdown cells relative to ALYREF with K171A mutant. Data show the mean ± S.D., *n* = 3. The *P*-values were calculated by a two-tailed unpaired Student’s *t-*test. **J** RNA stability assay showing *RABL6* (Left) and *TK1* (Right) mRNA half-life in T24 cells transfected with WT or K171A mutant plasmids after the knockdown of ALYREF. *n* = 3. The *P*-values were calculated by a two-tailed unpaired Student’s *t*-test. **K** Western blotting showing the protein expression of ALYREF, RABL6 and TK1 in control and shALYREF#3 T24 cells, which expressing WT ALYREF and K171A mutant ALYREF and were normalized by α-tubulin expression. **L** Western blotting assays showing the protein expression level of RABL6, TK1 and NSUN2 in siCTRL and siNSUN2 cells and were normalized by α-tubulin expression.

To further identify the potential mRNAs regulated by ALYREF, we performed RNA-seq analysis in control or ALYREF knockdown T24 cells. After knockdown of ALYREF, 143 mRNAs differentially expressed, including 94 downregulated mRNAs (NC reads >100, |Fold change | >1.5, *P*-value <0.05). Functional enrichment analysis using Kyoto Encyclopedia of Genes and Genomes (KEGG) indicated that regulated mRNAs by ALYREF are chiefly enriched in canonical cancer-related pathways (Fig. S4A), including TGF-β signaling, MAPK signaling, and NF-κB signaling, strongly supporting the oncogenic function of ALYREF in tumor progression. Among these 94 downregulated genes, 11 mRNA showed a significant reduction in m^5^C methylation after NUSN2 silencing in T24 cells, combined with previously transcriptome-wide RNA-BisSeq data of T24 cells [21] (Fig. 4A). We further combined RNA-BisSeq in HeLa cells from Yang et al. [14] and Huang et al. [12], and found 3 mRNAs showed a significant decrease in m^5^C methylation after NUSN2 silencing (Table S7). We next analyzed the ALYREF-RIP-BisSeq from Yang et al. [14] and identified that m^5^C methylated *RABL6* (chr9: 139702478) and *TK1* (chr17: 76170268) were located in ALYREF-RIP RNAs (Fig. 4B) (Table S8).

According to the RNA-BisSeq from Chen et al. [21], we found that knockdown of NSUN2, the m^5^C level of *RABL6* (chr9: 139702478) was reduced from 0.3528 to 0.1386, while the m^5^C level of *TK1* (chr17: 76170268) was reduced from 0.164 to 0. For further validation, we performed m^5^C-RIP- quantitative real-time polymerase chain reaction (qRT-PCR) and found that knockdown of NUSN2 substantially reduced the m^5^C level of *RABL6* and *TK1* (Fig. 4C). These results together demonstrated that *RABL6* contains m^5^C site in the 5′UTR (chr9: 139702478); *TK1* contains m^5^C site in the 3′UTR (chr17: 76170268). We next performed RNA immunoprecipitation-sequencing (RIP-seq) and RIP-qRT-PCR to identify ALYREF binding targets. ALYREF-Flag-RIP seq from Yang et al. [14] (Table S9) and our RIP-seq confirmed that ALYREF interacted with the m^5^C sites of *RABL6* and *TK1* mRNA (Fig. 4D and S4B). Then, we conducted RIP-qRT-PCR analysis by endogenous ALYREF to confirm the binding to targeted mRNAs. When ALYREF was depleted, the relative enrichment of *RABL6* and *TK1* mRNA was reduced (Fig. 4E). Through qRT-PCR assay, we found the expression of *TK1* and *RABL6* mRNA were dramatically reduced by ALYREF depletion (|log2FC | > 1, *P* <0.0001) (Fig. 4F). These results suggest *RABL6* and *TK1* are direct targets of NSUN2 and ALYREF mediated m^5^C methylation or recognition.

### ALYREF promotes *RABL6* and *TK1* splicing and maintains their stabilization

To unveil the biological significance of m^5^C methylation through ALYREF recognition, we purified ALYREF-bound proteins subjected to mass spectrometry analysis (Fig. 4G and S4C). The result showed that several spliceosome factors bound to ALYREF (Fig. S4D), such as SRSF3, PRPF3 and DHX16, indicating ALYREF may function in the regulation of mRNA splicing. We applied iREAD (intron REtention Analysis and Detector) [39] to analyze the reads of shCTRL and shALYREF#3 RNA-seq and found intron retention events in *RABL6* and *TK1* after ALYREF knockdown (Fig. S4E). We therefore investigated whether ALYREF affects the splicing of *RABL6* and *TK1* mRNA. The splicing efficiency was determined by qRT-PCR, whereas exon-intron pair amplifies premature isoform mRNA, exon-exon pair amplifies mature form mRNA. After knockdown of ALYREF, the splicing efficiency of *RABL6* and *TK1* was significantly decreased as measured by the ratio of spliced/ unspliced intermediates. Moreover, exogenous expression of WT ALYREF, but not the K171A mutant of ALYREF, restored the splicing efficiency of *RABL6* and *TK1* (Fig. 4H). Further analysis showed ALYREF knockdown reduced the level of mature *RABL6* and *TK1* mRNA, but did not affect the level of premature *RABL6* and *TK1*(Fig. S4F). Similarly, we found that NSUN2 knockdown did not affect the level of premature *RABL6* and *TK1*. However, the level of mature *RABL6* and *TK1* mRNA was downregulated in NSUN2 knockdown cells (Fig. S4G). As the improperly spliced mRNAs are retained in the nucleus for RNA quality check [40], therefore we determined whether ALYREF recognition of m^5^C methylated mRNA facilitated mRNA export. We isolated nuclear and cytoplasmic RNA fractions and quantified the quantity of *RABL6* and *TK1* in each fraction by qRT-PCR. We found that after depletion of ALYREF, *RABL6* and *TK1* mRNAs was retained in the nucleus. Overexpression of exogenous WT ALYREF, but not the K171A mutant of ALYREF, restored the proper export of *RABL6* and *TK1* mRNA (Fig. 4I). We further investigated the RNA stability of *RABL6* and *TK1* by ALYREF depletion. After treatment with actinomycin D, the stability of *RABL6* and *TK1* was strongly decreased by depletion of ALYREF, while this reduction could be rescued by exogenous WT ALYREF, but not the K171A mutant ALYREF (Fig. 4J). Luciferase reporter assays showed that ALYREF depletion substantially reduced the luciferase mRNA expression and activity of RABL6 with WT m^5^C site (RABL6-WT) and TK1 with WT m^5^C site (TK1-WT), but not RABL6 with mutant m^5^C site (RABL6-Mut) and TK1 with mutant m^5^C site (TK1-Mut) (Fig. S4H and S4I). In accordance with these results, RABL6 and TK1 protein expression were strongly diminished after ALYREF or NSUN2 depletion (Fig. 4K, L). Overexpression of exogenous WT ALYREF, but not the K171A mutant of ALYREF, restored RABL6 and TK1 protein expression (Fig. 4K). These results suggest that m^5^C methylation through ALYREF recognition facilitates splicing and maintain stabilization, which consequently leads to proper mRNA export and protein expression.

To further explore whether the K171A mutation impairs the binding of ALYREF to RNA in general, we performed RIP-qRT-PCR analysis to determine the general binding ability of ALYREF K171A mutant to RNA. We found that ALYREF WT binds to m^5^C sites of *RABL6*, while ALYREF K171A mutant showed lower level of binding ability to m^5^C sites of *RABL6*. Moreover, ALYREF WT and K171A mutants showed the similar binding ability to *RBM26* (chr13:79893003-79980390), *SLC39A9* (chr14:69865409-69929107), and *NUMB* (chr14:73741918-73925286), which don’t contain m^5^C sites from studies of Yang et al. [14], Huang et al. [12] and Chen et al. [21] (Fig. S4J). These data indicate that K171A mutation does not affect general binding ability of ALYREF to RNA.

### ALYREF enhances UCB pathogenesis in an m^5^C-dependent manner

**Fig. 5:**
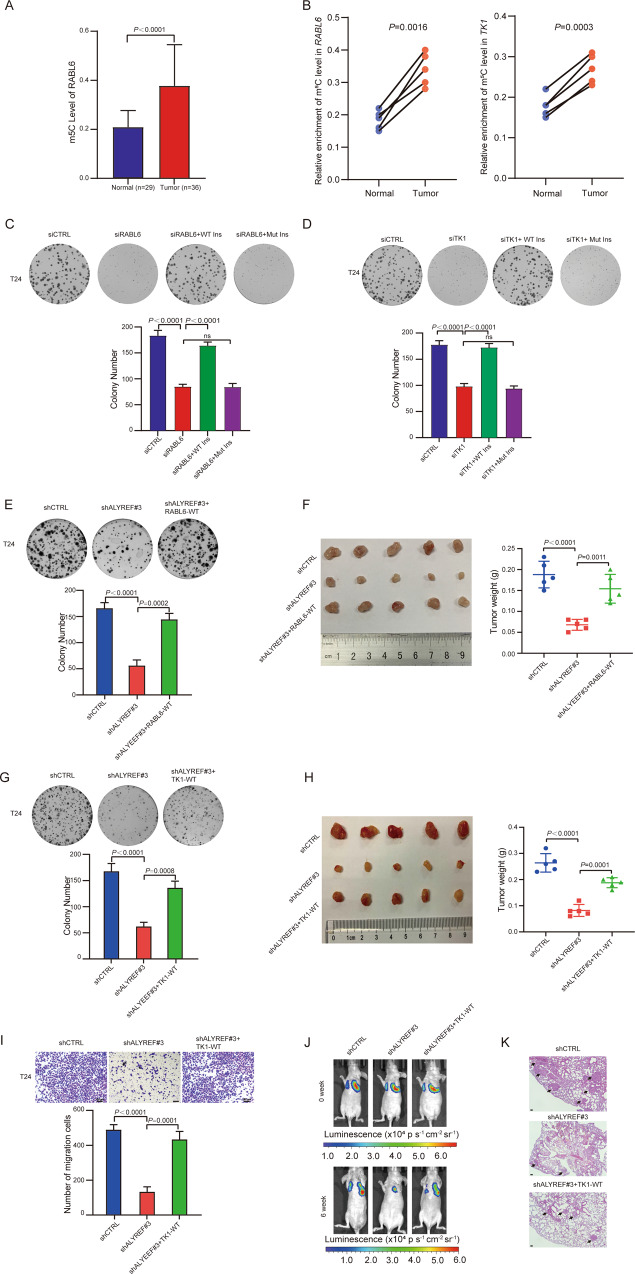
ALYREF enhances UCB pathogenesis in an m^5^C-dependent manner. **A** The m^5^C level of *RABL6* mRNA in 36 UCBs and in 29 adjacent non-neoplastic tissues from SYSUCC. Data represent the mean ± S.D. The *P*-values were calculated by a two-tailed unpaired Student’s *t*-test. **B** m^5^C-RIP-qRT-PCR showing the relative enrichment of m^5^C level of *RABL6* and *TK1* was upregulated in UCB. Left: m^5^C-RIP-qRT-PCR showing the relative enrichment of m^5^C level of *RABL6* in 5 pairs of UCB and normal tissues. Right: m^5^C-RIP-qRT-PCR showing the relative enrichment of m^5^C level of *TK1* in 5 pairs of UCB and normal tissues. Data represent the mean ± S.D., *n* = 5, and a two-tailed paired Student’s *t*-test was applied to determine the *P-*value. **C** Colony forming assay showing the effect of RABL6 with a WT m^5^C site on the restoration of cell growth in RABL6-knockdown cells relative to RABL6 with a mutated m^5^C site. Top: representative images of cell colonies; Bottom: histograms of colony numbers. Data show the mean ± S.D., *n* = 3. The *P*-values were calculated by a two-tailed unpaired Student’s *t*-test. **D** Colony forming assay showing the effect of TK1 with a WT m^5^C site on the restoration of cell growth in TK1-knockdown cells relative to TK1 with a mutated m^5^C site. Top: representative images of cell colonies; Bottom: histograms of colony numbers. Data show the mean ± S.D., *n* = 3. The *P-*values were calculated by a two-tailed unpaired Student’s *t*-test. **E** Colony forming assay showing the effect of RABL6 with a WT m^5^C site on the restoration of cell growth in ALYREF-knockdown cells. Top: representative images of cell colonies; Bottom: histograms of colony numbers. Data show the mean ± S.D., *n* = 3. The *P-*values were calculated by a two-tailed unpaired Student’s *t-*test. **F** Left: The subcutaneous xenograft model showing the effect of RABL6 with a WT m^5^C site on the restoration of subcutaneous tumor formation in ALYREF-knockdown cells. Right: statistical results for the mean tumor weight in different groups. Data show the mean ± S.D. The *P-*values were calculated by a two-tailed unpaired Student’s *t*-test. *n* = 5. **G** Colony forming assay showing the effect of TK1 with a WT m^5^C site on the restoration of cell growth in ALYREF-knockdown cells. Top: representative images of cell colonies; Bottom: histograms of colony numbers. Data show the mean ± S.D., *n* = 3. The *P*-values were calculated by a two-tailed unpaired Student’s *t*-test. **H** Left: The subcutaneous xenograft model showing the effect of TK1 with a WT m^5^C site on the restoration of subcutaneous tumor formation in ALYREF-knockdown cells. Right: Statistical results for the mean tumor weight in different groups of the subcutaneous xenograft model. Data indicates the mean ± S.D. The *P*-values were calculated by a two-tailed unpaired Student’s *t-*test. *n* = 5. **I** Migration assay showing the effect of TK1 with a WT m^5^C site on the restoration of cell migration in ALYREF-knockdown cells. Top: representative images of migration cells; Bottom: histograms of migration cell numbers. Data show the mean ± S.D., *n* = 3. The *P*-values were calculated by a two-tailed unpaired Student’s *t*-test. **J** The lung metastasis model showing the effect of TK1 with a WT m^5^C site on the restoration of tumor metastasis in ALYREF-knockdown cells. Representative bioluminescence images are shown at 0 and the 6th week after injection. **K** Hematoxylin-eosin staining and metastatic nodules (indicated by arrows) in lung tissues from different groups at the 6th week. Scale bars: 100 µm.

To further determine the pathological significance of m^5^C methylation at *RABL6* and *TK1* mRNA, we analyzed previous RNA-BisSeq from SYSUCC cohort. The result indicated that the m^5^C level of *RABL6* was higher in tumor tissue than that in normal tissues (Fig. 5A). We then collected 5 pairs of UCB and normal tissues and extracted RNA to conduct m^5^C-RIP-qRT-PCR. The results showed an m^5^C hypermethylation of *RABL6* and *TK1* in tumors compared to the normal tissues (Fig. 5B). These results indicated potential oncogenic roles of *RABL6* and *TK1* m^5^C methylation in UCB progression. We next constructed siRNA-insensitive RABL6 and TK1 expression plasmids either with WT m^5^C-site (WT Ins) or mutated m^5^C-site (Mut Ins) to investigate the pathological significance of m^5^C methylation at *RABL6* and *TK1* mRNA (Fig. S5A–D). We next explored whether the mutant could affect the m^5^C level of *RABL6* and *TK1*. We conducted m^5^C-RIP-qRT-PCR in T24 cells transferred RABL6-WT, RABL6-Mut, TK1-WT and TK1-Mut, respectively. As showed in Fig. S5E, the relative enrichment of m^5^C level was reduced significantly in RABL6-Mut and TK1-Mut cells compared with RABL6-WT and TK1-Mut, respectively. The colony-formation assay showed that knockdown of RABL6 or TK1 could significantly reduce colony-formation ability, and this reduction could be recovered by RABL6 or TK1 with WT m^5^C-site but not by m^5^C site- mutated RABL6 or TK1 (Figs. 5C, D and S5F, G). These findings suggest hypermethylated *RABL6* and *TK1* promote UCB pathogenesis.

To further investigate the functional correlation between ALYREF and RABL6 and TK1, we conducted rescue experiments respectively. We overexpressed RABL6 with WT m^5^C-site in ALYREF-knockdown T24 cells (Fig. S5H). After knockdown of ALYREF, the inhibited colony formation and subcutaneous tumor formation were partially rescued by expressing RABL6 with WT m^5^C-site (Fig. 5E and F). Migration assays showed that RABL6 could not rescued reduced tumor cell migration capacity caused by ALYREF (Fig. S5I). Next, we overexpressed TK1 with WT m^5^C-site in ALYREF-knockdown T24 cells (Fig. S5J). After knockdown of ALYREF, the inhibited colony formation and subcutaneous tumor formation were partially rescued by expressing TK1 with WT m^5^C-site (Fig. 5G and H). Migration assays and tail-vein injection metastasis assays showed that the inhibited cell migration and lung metastasis were partially rescued by expressing TK1 with WT m^5^C-site (Fig. 5I–K and Fig. S5K and S5L).

Taken together, these findings demonstrate that ALYREF promote UCB pathogenesis in an m^5^C-dependent manner.

### ALYREF recognizes hypermethylated m^5^C site of *NSUN2*, resulting in NSUN2 upregulation in UCB

**Fig. 6:**
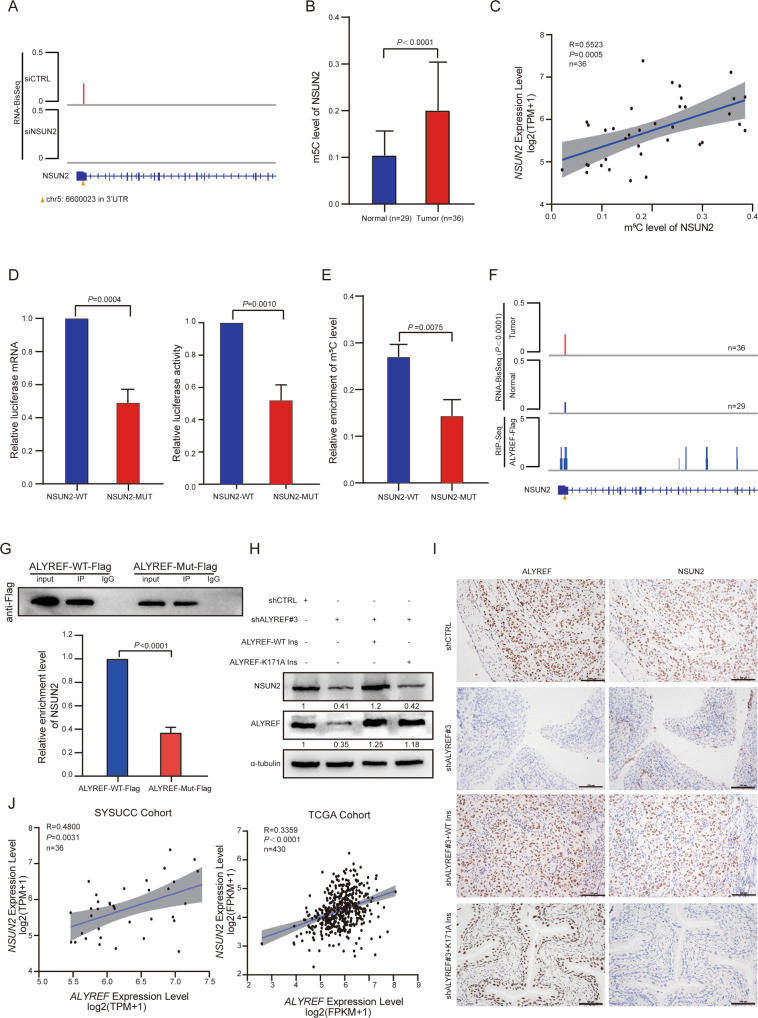
ALYREF recognizes hypermethylated m^5^C site of *NSUN2*, resulting in NSUN2 upregulation in UCB. **A** Integrative- genomics-viewer tracks representing the methylated level of m^5^C sites in NSUN2 when NSUN2 was silenced (the methylated level is 0.213 for siCTRL and empty for siNSUN2, respectively). The triangle represents the m^5^C site in NSUN2 (chr5: 6600023). **B** The m^5^C level of NSUN2 in 36 UCBs and in 29 adjacent normal tissues from SYSUCC. A two-tailed unpaired Student’s *t*-test was applied to determine the *P*-value. **C** Pearson correlation analysis showing the association between *NSUN2* mRNA expression and its m^5^C level in 36 UCBs of SYSUCC. Shaded regions represent the 95% confidence interval. **D** Luciferase reporter assay showing the luciferase mRNA (Left) and activity (Right) level of NSUN2-wild-type m^5^C site containing plasmid and NSUN2 mutated m^5^C site containing plasmid in T24 cells. Data represent the mean ± S.D., *n* = 3. The *P-v*alue was determined by a two-tailed unpaired Student’s *t-*test. **E** The relative enrichment of m^5^C level in wild-type NSUN2 containing m^5^C-site compared with m^5^C-site mutant NSUN2. Data represent the mean ± S.D., *n* = 3, and a two-tailed unpaired Student’s *t-*test was applied to determine the *P-*value. **F** Integrative- genomics-viewer tracks representing the read coverage of NSUN2 in ALYREF-Flag RIP-seq data and the m^5^C levels of 36 UCB and 29 adjacent non-neoplastic tissues from SYSUCC. The triangle indicates the m^5^C site (chr5: 6600023) in NSUN2. **G** Upper panel: Western blotting shows Flag IP efficiency between ALYREF WT and K171A mutant. Bottom panel: Relative enrichment representing *NSUN2* mRNA levels associated with ALYREF compared to an input control. IgG antibody used as a control. Data show the mean ± S.D., *n* = 3. The *P*-values were calculated by a two-tailed unpaired Student’s *t*-test. **H** Western blotting assays showing the expression level of NSUN2 and ALYREF in control and shALYREF#3 T24 cells, which expressing WT ALYREF and the K171A mutant and were normalized by α-tubulin expression. **I** IHC staining assays of mice bladder slices from orthotopic xenograft models showing the effect of ALYREF with a WT m^5^C site on the restoration of the ALYREF (Left column) and NSUN2 (Right column) expression in ALYREF-knockdown cells relative to ALYREF with K171A mutant. Scale bars, 100 μm. **J** Pearson correlation analysis showing the association between *NSUN2* and *ALYREF* mRNA expression in the SYSUCC cohort (Left, *n* = 36) and TCGA cohort (Right, *n* = 430). Shaded regions showed the 95% confidence interval.

From our previous RNA-BisSeq data in T24 cell, we identified an m^5^C methylation site located in the 3′UTR of *NSUN2* (chr5: 6600023). Knockdown of NSUN2 significantly reduced the m^5^C methylation level of NSUN2 (Fig. 6A). This m^5^C site was accordance with the result from the RNA-BisSeq data from Huang et al. [12] and RNA-BisSeq data from Yang et al. [14] (Table S7). RNA-BisSeq derived from SYSUCC cohort showed that the m^5^C methylation level of NSUN2 in UCB tumors was higher than that in normal tissues (Fig. 6B). Additionally, the mRNA expression of *NSUN2* was positively associated with the m^5^C level of *NSUN2* mRNA in 36 UCB tissues (Fig. 6C), indicating that NSUN2 expression may be regulated by its mRNA m^5^C methylation level. To test this hypothesis, we constructed luciferase reporter carried NSUN2 with WT m^5^C site or NSUN2 with mutated m^5^C site. As expected, the luciferase mRNA and activity level of NSUN2 containing WT m^5^C site plasmid was significantly higher compared to NSUN2 containing mutated m^5^C site plasmid, suggesting that NSUN2 expression requires m^5^C methylation at its 3′UTR. (Fig. 6D). We then conducted m^5^C-RIP-qRT-PCR to analyze the relative enrichment of m^5^C level in *NSUN2* mRNA with a WT or mutant m^5^C-site. The relative enrichment of m^5^C level was reduced significantly in NSUN2-Mut cells compared with NSUN2-WT (Fig. 6E). To further identify the reader of m^5^C methylation at *NSUN2*, we found that the ALYREF-RIP-BisSeq data from Yang et al. [14] showed that m^5^C methylated NSUN2 (chr5: 6600023) were located in ALYREF-RIP RNAs (Table S8). Our ALYREF RIP-seq data (Fig. 6F) and ALYREF RIP-seq data of Yang et al. [14] (Table S9) showed that the binding site of ALYREF on *NSUN2* mRNA coincided well with the m^5^C site of *NSUN2*. The specific binding was further confirmed by RIP-qRT-PCR assay (Fig. 6G). Western blotting assays indicated that NSUN2 expression was reduced when ALYREF was knocked down, and the reduction could be rescued by WT but not K171A-mutant ALYREF (Fig. 6H). In addition, by IHC analysis of mice bladder slices from orthotopic xenograft model, the downregulation of NSUN2 was correlated with ALYREF knockdown. WT, but not K171A mutant ALYREF could restore the expression of NSUN2 (Fig. 6I). These data together indicate that ALYREF recognizes hypermethylated m^5^C site of *NSUN2*, resulting in NSUN2 upregulation in UCB. Clinically, RNA-seq analysis of the SYSUCC cohort and TCGA cohort showed that *NSUN2* and *ALYREF* levels were positively correlated in UCB (Fig. 6J), suggesting that NSUN2-ALYREF cross-regulation is a bona fide mechanism in UCB progression, contributing to the homeostatic control of RNA m^5^C methylation.

### ALYREF-RABL6-TK1 m^5^C related axis predicts poorest OS in UCB

**Fig. 7:**
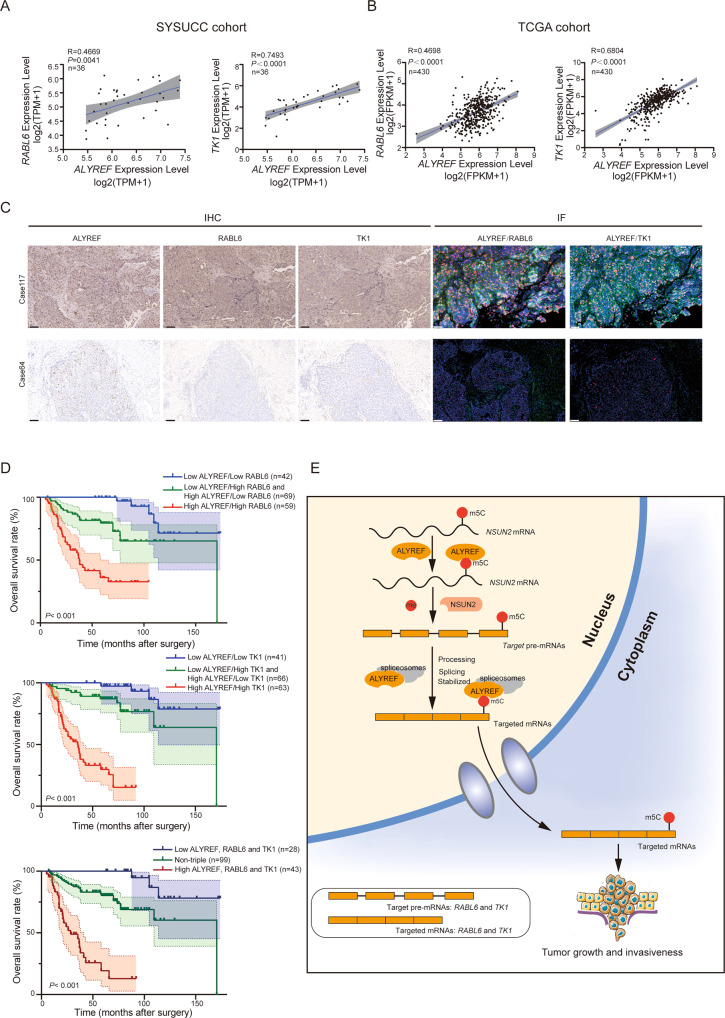
ALYREF-RABL6-TK1 m^5^C related axis predicts poorest overall survival in UCB. **A** Pearson correlation analysis showing the association between *RABL6, TK1* and *ALYREF* mRNA expression in the SYSUCC cohort. *n* = 36. Shaded regions showed the 95% confidence interval. **B** Pearson correlation analysis showing the association between *RABL6, TK1* and *ALYREF* mRNA expression in TCGA cohort. *n* = 430. Shaded regions showed the 95% confidence interval. **C** Representative IHC staining and double immunofluorescence staining images of ALYREF, RABL6 and TK1 in two UCB tissues with high (the first row) or low expression (the second row). Blue (DAPI) = cell nuclei, red (Cyanine 3) = ALYREF, green (Alexa 488) = RABL6/TK1. Scale bars, 100 μm. **D** Kaplan–Meier analysis of data of 170 UCB patients from SYSUCC showing the correlation between different expression patterns of ALYREF/ RABL6 (Top, *P* <0.001) and OS, the correlation between different expression patterns of ALYRREF/TK1 (Medium, *P* <0.001) and OS, and the correlation between different expression patterns of ALYREF/ RABL6/ TK1 (Bottom, *P* <0.001), and OS. The *P-*values were calculated by a log-rank test. **E** Schematic illustration showing that m^5^C dependent cross-regulation between nuclear reader ALYREF and writer NSUN2 promotes urothelial bladder cancer malignancy through facilitating mRNAs splicing and stabilization.

Next, we examined expression levels of ALYREF and downstream m^5^C-methylated proteins (RABL6 and TK1) in UCB tissue samples from SYSUCC and TCGA cohort. From RNA-seq analysis, the expression levels of *RABL6* and *TK1* mRNA are positively associated with the levels of *ALYREF* mRNA in SYSUCC and TCGA cohort. (Fig. 7A, B). The IHC analysis and double immunofluorescence staining showed that the expression levels of RABL6 and TK1 were positively associated with that of ALYREF in UCBs (Fig. 7C and Fig. S5M). Furthermore, subgroup of individuals with UCBs was classified to investigate the relationship between ALYREF-m^5^C-related proteins (RABL6 and TK1) and survival rate. Notably, high levels of ALYREF and high levels of RABL6 or TK1 were significantly associated with poorer OS. Triple high expression of ALYREF, RABL6 and TK1 was correlated with the poorest OS in SYSUCC cohort (Fig. 7D). Collectively, these data suggest that ALYREF- RABL6-TK1 m^5^C-related axis is involved in UCB aggressiveness (Fig. 7E), highlighting its potential as a diagnostic marker and therapeutic target for UCBs.

## Discussion

Epigenetic modifications play essential roles in gene regulation, environmental interactions and cancers [41]. m^6^A modification has been identified as an important factor in the determination of mammalian cell fate transition, embryonic stem cell differentiation and tumorigenesis [42]. Several studies have suggested that m^6^A regulators were upregulated in cancers and m^6^A modification promotes the development of tumors [43–45]. Since RNA modifications were controlled by regulators, abnormal expression of these regulators may cause tumorigenesis or cancer progression. As a kind of RNA modifications, m^5^C plays an important role in cancer tumorigenesis. In this study, by integration of RNA-seq data from SYSUCC and TCGA, we found that m^5^C regulators including ALYREF are consistently upregulated in UCB compared to normal tissues, and upregulated ALYREF is positively associated with UCB patients’ poorer OS. m^5^C regulators were positively associated with multiple oncogenic pathways. These results supported that ALYREF may play an essential part in bladder cancer.

We applied the model of patient-derived organoids to explore the function of ALYREF. Several studies demonstrated that organoid models maintain key features from their parental tumors, such as genetic and phenotypic heterogeneity, allowing them to be used for a wide spectrum of applications [36, 38]. In addition, organoids can be established and expanded with high efficiency from primary patient material [46]. Moreover, organoid models showed improved resemblance to the original tumor compared to 2D cultured cancer cell lines [37, 38]. Therefore, we demonstrated that patient-derived organoids serve as an ideal cell model to study tumor pathogenesis. Considering organoid cultures bridge the gap between in vitro 2D cancer cell line cultures and in vivo parental tumors, we thus applied organoids a promising tool to further explored the biological function of ALYREF.

Combined with RNA BisSeq data of Yang et al. [14], Huang et al. [12] and our previous reports, we confirmed *RABL6* and *TK1* are direct targets of NSUN2. The ALYREF-RIP-BisSeq data from Yang et al. [14] identified m^5^C methylated *RABL6*, *TK1* and *NSUN2* were enriched in ALYREF-RIP RNAs. The ALYREF-RIP-seq from Yang et al. [14] showed ALYREF interacted with m^5^C methylation sites of *RABL6*, *TK1* and *NSUN2*. These results suggested that the m^5^C sites of *RABL6*, *TK1* and *NSUN2* were true recognized by ALYREF and regulated by NSUN2 methylation. It has been reported that NSUN2 and NSUN6 play important roles in Type I or Type II-modified m^5^C in mRNAs [7, 12, 13]. To further analyze RNA secondary structure of m^5^C site of *TK1*, *RABL6* and *NSUN2*, we extracted the upstream and downstream 25 bp sequences of the m^5^C sites (Table S10) and used RNAfold tool (http://rna.tbi.univie.ac.at/cgi-bin/RNAWebSuite/RNAfold.cgi) to complete this prediction. The analysis showed that the m^5^C site of *TK1* containing a downstream G-rich triplet motif, which may be similar to Type I modified m^5^C described by Huang et al. [14]. RNAfold prediction revealed *TK1* m^5^C site have a tRNA-like structure. However, the m^5^C sites of *RABL6* and *NSUN2* did not contain a downstream G-rich triplet motif, and did not show a tRNA-like structure. Therefore, based on these results and our findings, we demonstrated that m^5^C sites, which represent tRNA-like structures or tRNA-unlike structures, were both regulated by NSUN2.

RABL6 and TK1 are well-known oncogenes and promote tumor proliferation in many types of cancers [47–50]. Xu et al. [51] found that circTMC5 sponged miR-361-3p to up-regulate RABL6 expression to promotes gastric cancer. Gandhi et al. [52] showed lincNMR-YBX1 axis regulated TK1 expression by binding its promoter regions. In our study, the mutant of m^5^C site at *RABL6* and *TK1* could reduce proliferative capacity of tumors. It is as well reported that the m^5^C at a particular mRNA position may affect tumor stage. Sun et al. [26] found NSUN2-mediated m^5^C modification of H19 lncRNA is associated with poor differentiation of HCC. By applying bisulfite-PCR pyrosequencing, they found the methylation level at the H19 C986 site in HCC tissues was significantly higher than that in matched non-cancerous liver tissues. The m^5^C methylation level of H19 RNA in HCC patients are significantly associated with the differentiation stages of tumors (*P* <0.001). Our results propose a novel m^5^C-modification-dependent mechanism of RABL6 and TK1 expression, which contributes in UCB progression.

The removal of introns by splicing is an important step of precursor mRNA process, which frequently altered in tumors [53]. Splicing abnormalities can result in tumor proliferation [54], progression and invasion [55]. Epigenetic modifications including m^6^A modification, participated in mRNA splicing to regulate tumorigenesis and development. m^6^A writers like METTL16 [56] and METTL13 [57], m^6^A readers like YTHDC1 [58], HNRNPA2B1 [59], and m^6^A erasers like FTO [60] were reported to mediate mRNA splicing to control tumors. Specifically, splicing factors like SRSF3, which interacted with YTHCD1 to promote mRNA splicing and nucleus export of m^6^A-modified mRNAs, was also found binding to ALYREF in our study and from Khan et al. [61] (Table S11). In addition, TREX complex have been found to bind with endogenous ALYREF from our study and from Khan et al. [61] (Table S11). Similarly, Mendel et al. [62] found that m^6^A modification was deposited on the 3′ splice site of the S-adenosylmethionine synthetase pre-mRNA, which inhibited proper splicing and protein production. We firstly reported that m^5^C reader ALYREF promoted UCB malignancy through regulating mRNA splicing via recruiting spliceosome to targeted hypermethylated mRNAs.

Little has been known about the cross-regulations among mRNA methylation regulators. Several studies showed cross-regulation between m^6^A regulators. Liu et al. [63] demonstrated that the expression of m^6^A writers was positively correlated with their m^6^A variation; additionally, conserved m^6^A peaks of m^6^A regulators were observed in all human tissues, suggesting that the transcripts of the m^6^A modification machineries are also susceptible to epitranscriptomic regulation. Panneerdoss et al. [64] revealed that the collaboration among METTL14-ALKBH5-YTHDF3 (writer-eraser-reader) sets up the m^6^A threshold to regulate the stability of target proliferation-specific gene, resulting in tumor progression. In the current study, we firstly demonstrated that ALYREF recognizes hypermethylated m^5^C site of *NSUN2*, resulting in NSUN2 upregulation in UCB. Integration of RNA-BisSeq and RNA-seq in UCB cell and tumor samples, we found that the m^5^C level of *NSUN2* mRNA was positively associated with *NSUN2* mRNA expression in SYSUCC cohort, suggesting that NSUN2 expression is regulated by its mRNA m^5^C methylation level. RIP-seq demonstrated that ALYREF recognizes hypermethylated m^5^C site of NSUN2, resulting in NSUN2 upregulation in UCB. Together, our study revealed that NSUN2-ALYREF cross-regulation is a bona fide mechanism in UCB progression, contributing to the homeostatic control of RNA m^5^C methylation.

In summary, our study underlines the significance of m^5^C methylation in human UCB. We demonstrate that ALYREF enhances proliferation and invasion of UCB cells in an m^5^C-dependent manner. ALYREF controls UCB malignancies through promoting hypermethylated *RABL6* and *TK1* mRNA for splicing and stabilization. Moreover, ALYREF recognizes hypermethylated m^5^C site of *NSUN2*, resulting in NSUN2 upregulation in UCB. Clinically, triple high expression of ALYREF/RABL6/TK1 axis predicts the poorest survival. Our study unveils a novel m^5^C dependent cross-regulation between nuclear reader ALYREF and m^5^C writer NSUN2 in activation of hypermethylated m^5^C oncogenic RNA, which consequently leads to tumor progression. These findings provide profound insights into therapeutic strategy for the disease.

## Materials and methods

### Patients and tissue sample collection

Protein samples collected from UCBs and adjacent non-neoplastic tissues of 10 patients who underwent radical cystectomy at SYSUCC were applied for western blotting analyses (Table S1).

A total of 170 UCBs and 30 adjacent non-neoplastic tissues from 170 UCB cases who underwent radical cystectomy from 2005 to 2016 at SYSUCC were used in the IHC analyses (Table S2). The TNM classification and tumor grades were defined in accordance with the eighth edition of the Union for International Cancer Control and the World Health Organization, respectively. Patients were followed up regularly depending on the guidelines. OS was defined as the time from treatment to the date of death due to any cause. After formalin fixation, all samples from these patients were subjected to paraffin-embedding and pathological diagnosis.

For the organoid model, the UCB tissue was collected from a UCB patient who underwent radical cystectomy and had a pathological diagnosis of UCB from SYSUCC (Table S3).

For the m^5^C-RIP-qRT-PCR, the 5 pairs of UCB and normal tissues were collected from patients receiving radical cystectomy and had a pathological diagnosis of UCB from SYSUCC (Table S4).

### Cell cultures

The cell lines used in our study, including SV-HUC-1, T24, UM-UC-3, TCC-SUP, 293 T cell lines, were obtained from American Type Culture Collection. RPMI-1640 medium (Invitrogen, Carlsbad, USA) containing 10% fetal bovine serum (HyClone, USA) was used to culture T24 cells. Other cell types were maintained in DMEM (Invitrogen, Carlsbad, USA) with 10% fetal bovine serum. A humidified incubator at 37 °C with 5% CO_2_ was provided for culturing cells. Cell lines were authenticated by short tandem repeat profiling and were tested free of mycoplasma contamination using PCR with TaKaRa PCR Mycoplasma Detection Set. All cell lines were cultured within 10 passages.

### Western blotting

Extracted proteins were dissolved in 1× SDS and then resolved by SDS-PAGE. After transfer to a PVDF membrane (Millipore, Massachusetts, USA), the membrane was incubated at 4 °C overnight with primary antibodies and room temperature for 1 h with secondary antibodies. The signals on the membranes were showed by an enhanced chemiluminescence kit (Tanon, Shanghai, China). The primary antibodies used for western blotting in our study were as follows: rabbit polyclonal anti-NSUN3 (Abclonal, Cat#: A12892; 1:1000), rabbit polyclonal anti-NSUN5 (Proteintech, Cat#:15449-1-AP; 1:1000), rabbit polyclonal anti-TET2 (Proteintech, Cat#: 21207-1-AP; 1:1000), rabbit polyclonal anti-TET3 (Abclonal, Cat#: A18319; 1:1000), rabbit polyclonal anti-ALYREF (Cell Signaling Technology, Cat#: 12655; 1:1000), rabbit polyclonal anti-NSUN2 (Proteintech, Cat#: 20854-I-AP; 1:5000), rabbit polyclonal anti-Flag-HRP (Cell Signaling Technology, Cat#: 2368 S; 1:1000), anti-α-tubulin (Beyotime, Cat#: AF0001; 1:1000), anti-TK1 (Proteintech, Cat#: 67787-1-Ig; 1:2000), and anti-RABL6 (Proteintech, Cat#: 20848-1-AP; 1:500).

### Immunohistochemistry

The obtained organs and tumors were formalin-fixed and paraffin-embedded. Then, 4-µm thick tissue sections were cut for IHC staining. Sections for IHC analysis were first heated at 65 °C for 2 h, deparaffnized in xylene and hydrated in graded alcohol. Endogenous peroxidase activity was inhibited in 3% hydrogen peroxide. Slides were incubated in Ethylenediaminetetraacetic Acid (EDTA) buffer (pH 8.0) for 5 min to retrieve antigen. After blocking nonspecific binding in 10% normal goat serum, primary antibodies for IHC were added for incubation overnight at 4 °C. Before staining with DAB staining solution and restaining with hematoxylin, the slides were incubated with secondary antibodies for 30 min at 37 °C. Seventy percent ethyl alcohol containing 0.1% hydrochloric acid was used to polarize the slides for 10 s.

Evaluation criteria including staining intensity and the positively stained area were applied for IHC staining. Staining intensity was divided into 0, 1, 2, and 3, which indicated no, weak, moderate and strong staining, respectively. The grades for positively stained cells included 1, 2, 3, and 4, which indicated a positively stained area of <10%, 10%–40%, 40%–70% and >70%, respectively. The immunoreactivity score combining the staining intensity and positively stained area scores was calculated by two independent pathologists who were blinded to the clinicopathological information. The primaries antibodies for IHC used in our study were as follows: rabbit polyclonal anti-ALYREF (Cell Signaling Technology, Cat#: 12655; 1:200), rabbit polyclonal anti-NSUN2 (Proteintech, Cat#: 20854-I-AP; 1:200), mouse monoclonal anti-TK1 (Proteintech, Cat#: 67787-1-Ig; 1:250), mouse polyclonal anti-RABL6 (Abnova, Cat#: H00055684-A01; 1:200).

### RNA interference

Short hairpin RNAs (shRNAs) used for ALYREF knockdown were acquired from GeneCopeia (Guangzhou, China), while short interfering RNAs (siRNAs) for NUSN3, NSUN5, TET2, TET3, NSUN2, TK1, and RABL6 knockdown were purchased from RIBOBIO (Guangzhou, China). Table S5 shows the sequences which are targeted for siRNAs and shRNAs.

Further materials and methods were shown in supplementary information.

### Statistical analysis

Statistical analysis was conducted with SPSS version 23.0 (IBM Corp., Armonk, NY, USA). Statistics are shown as the means ± SD. For analysis of the SYSUCC cohort, the differential expression of genes between UCB and normal tissues was analyzed by two-sided *t*-tests and the heatmap presenting the difference was generated by the R- package “Heatmap”. To analyze correlations among genes in the TCGA and SYSUCC cohorts, Spearman’s correlation analysis was applied. The Kaplan–Meier method and the log-rank test were conducted for survival analysis. The statistical significance between experimental groups were determined by two-sided *t*-tests or two-way ANOVA. The composition ratios were analyzed by the chi-square test. All experiments were independently conducted at least three times with similar results.

## Supplementary information

Supplementary Figure 1

Supplementary Figure 2

Supplementary Figure 3

Supplementary Figure 4

Supplementary Figure 5

Supplementary table 2

The supplementary information

Original Data File

The reproducibility checklist

## Data Availability

All data generated or analyzed during this study are included in this published article and its supplementary information files and supplementary figure 6. Additional data associated with this paper may be acquired from the corresponding author on reasonable request. The RNA-Seq data is deposited in the SRA database, and the Bioproject number is PRJNA765965.
