# Supplementary figures and images for: m5C-dependent cross-regulation between nuclear reader ALYREF and writer NSUN2 promotes urothelial bladder cancer malignancy through facilitating RABL6/TK1 mRNAs splicing and stabilization
Source: Cell Death Dis. 2023 Feb 18;14(2):139. doi: 10.1038/s41419-023-05661-y (PMC9938871; doi:10.1038/s41419-023-05661-y)

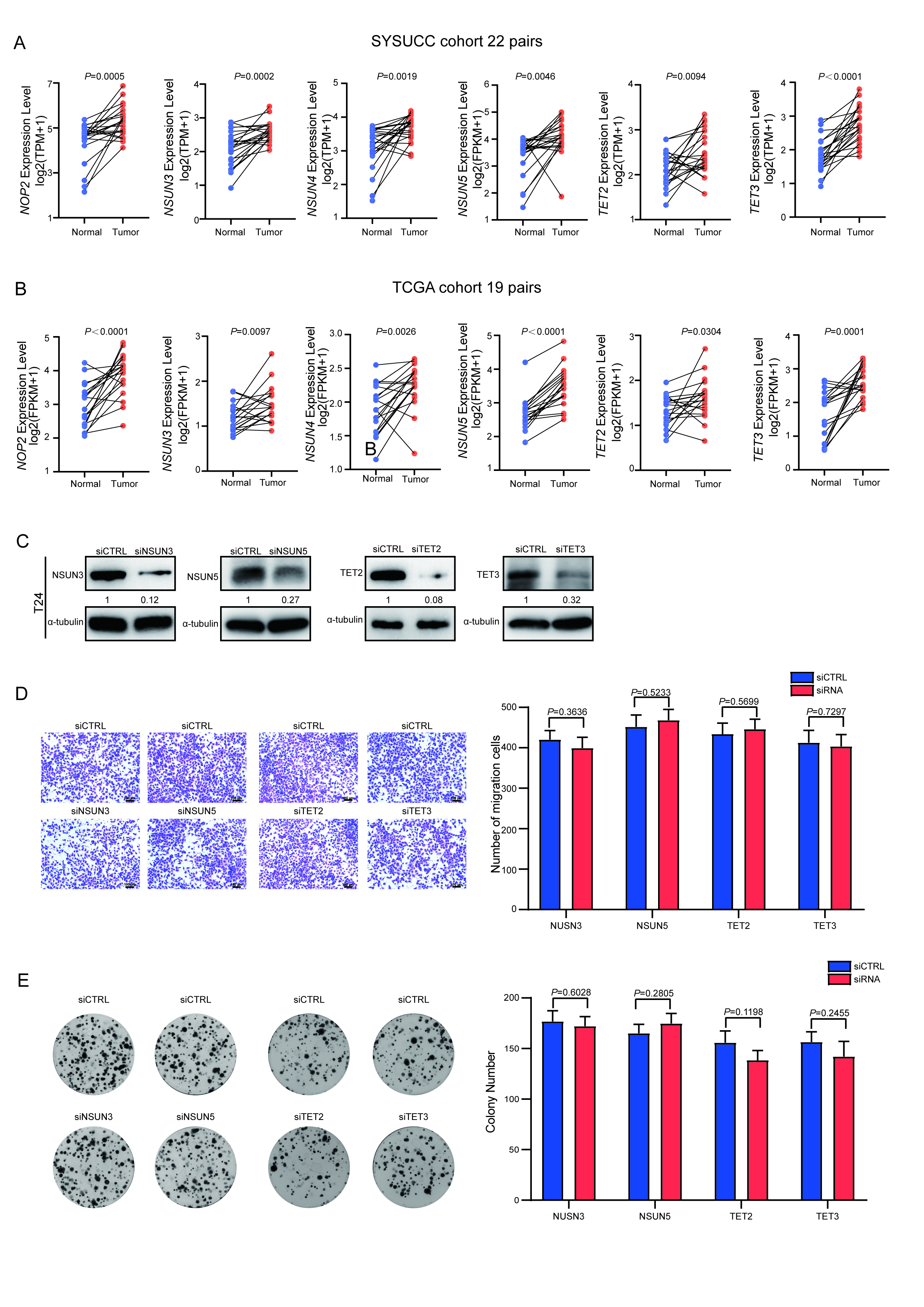

Supplement: Supplementary file 1 — Supplementary Figure 1 [file 41419_2023_5661_MOESM1_ESM.tif]

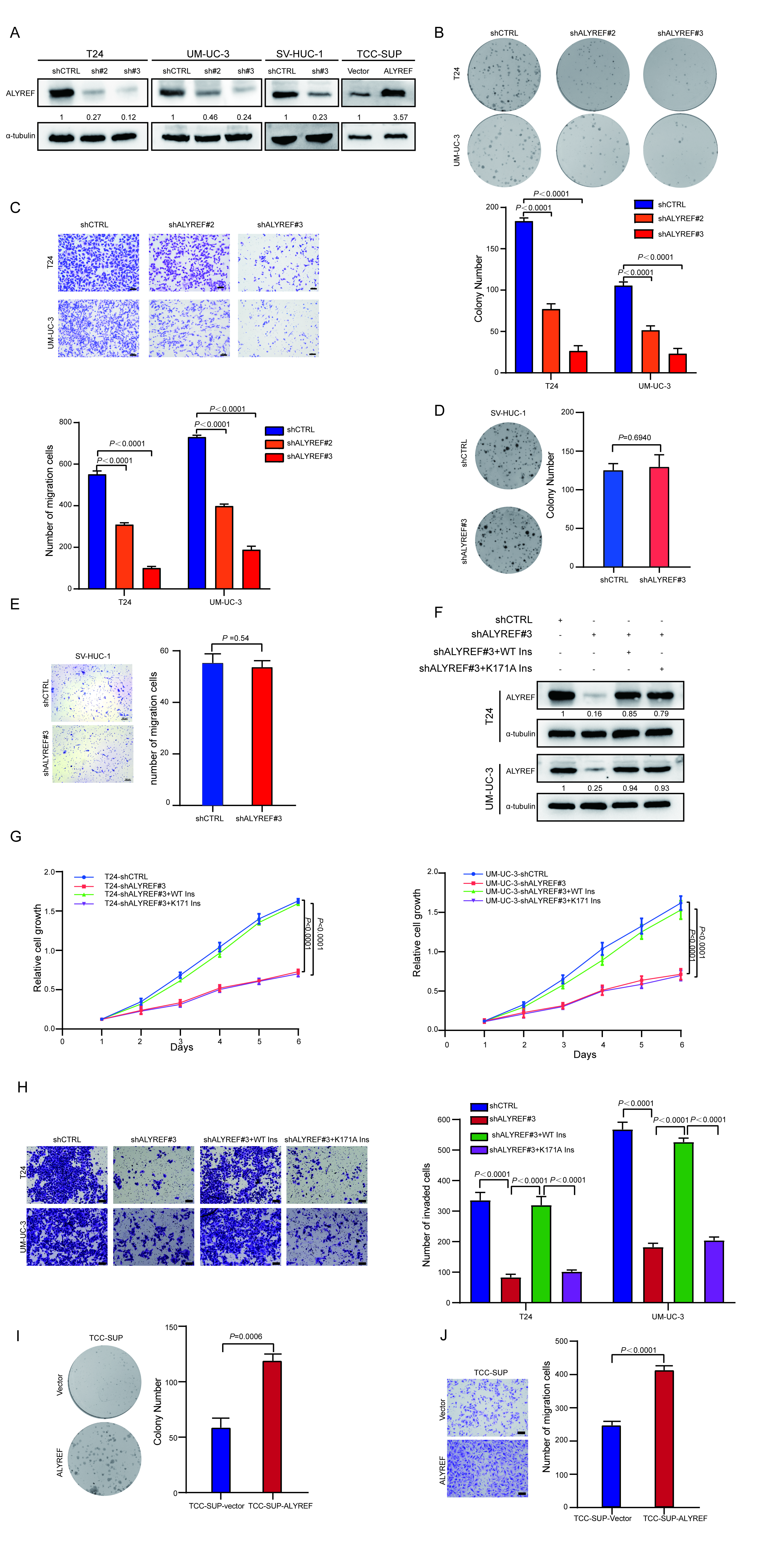

Supplement: Supplementary file 2 — Supplementary Figure 2 [file 41419_2023_5661_MOESM2_ESM.tif]

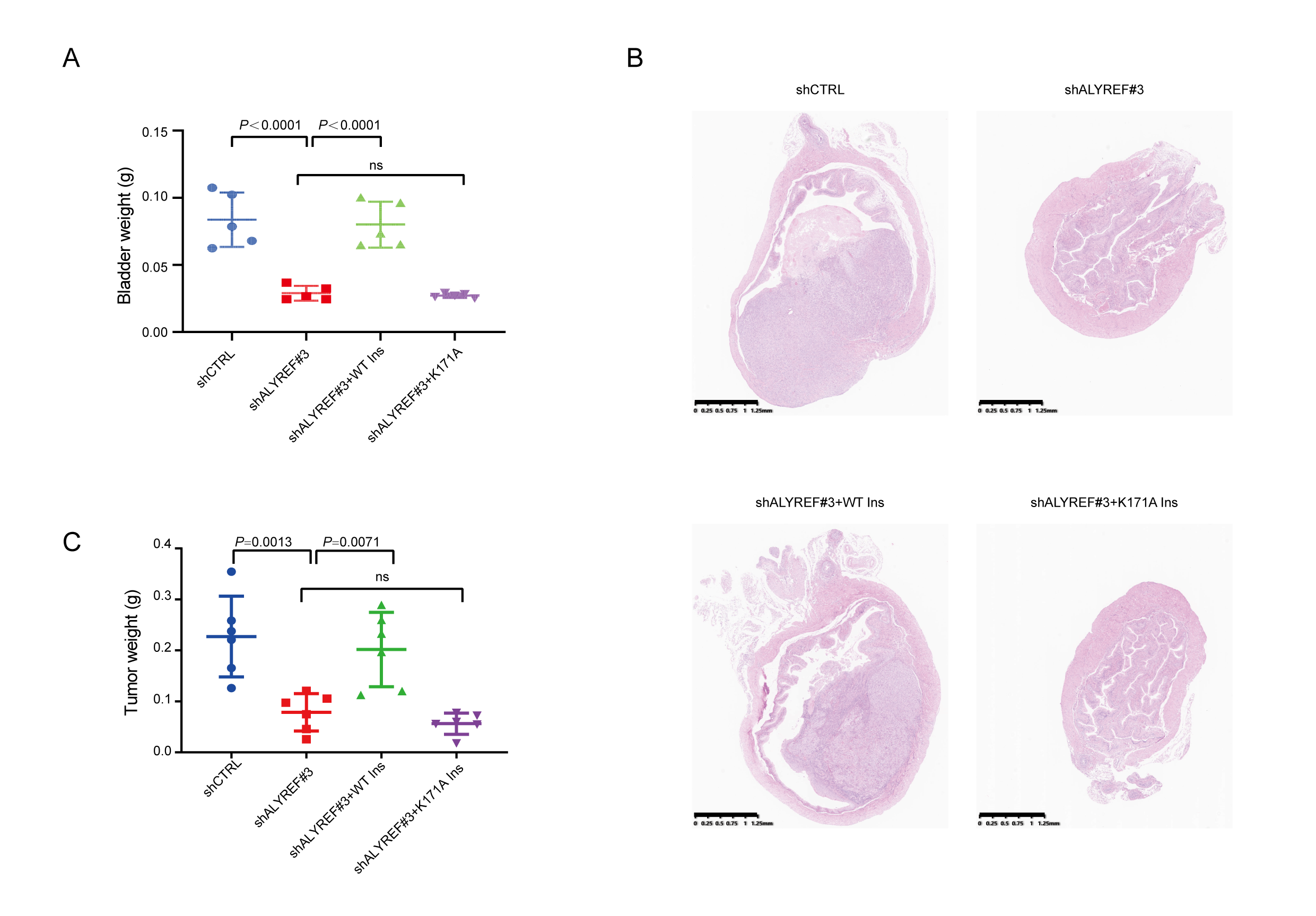

Supplement: Supplementary file 3 — Supplementary Figure 3 [file 41419_2023_5661_MOESM3_ESM.tif]

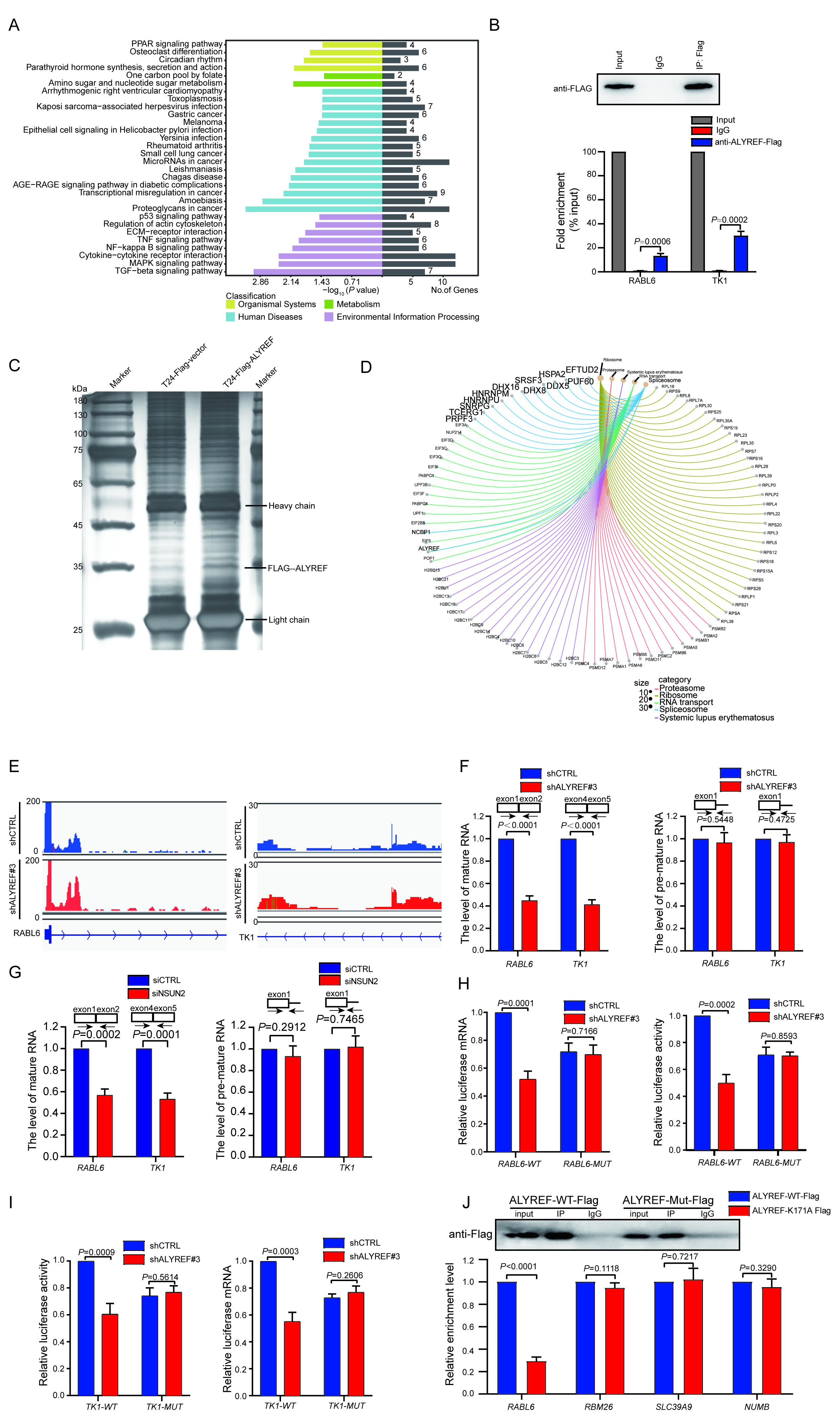

Supplement: Supplementary file 4 — Supplementary Figure 4 [file 41419_2023_5661_MOESM4_ESM.tif]

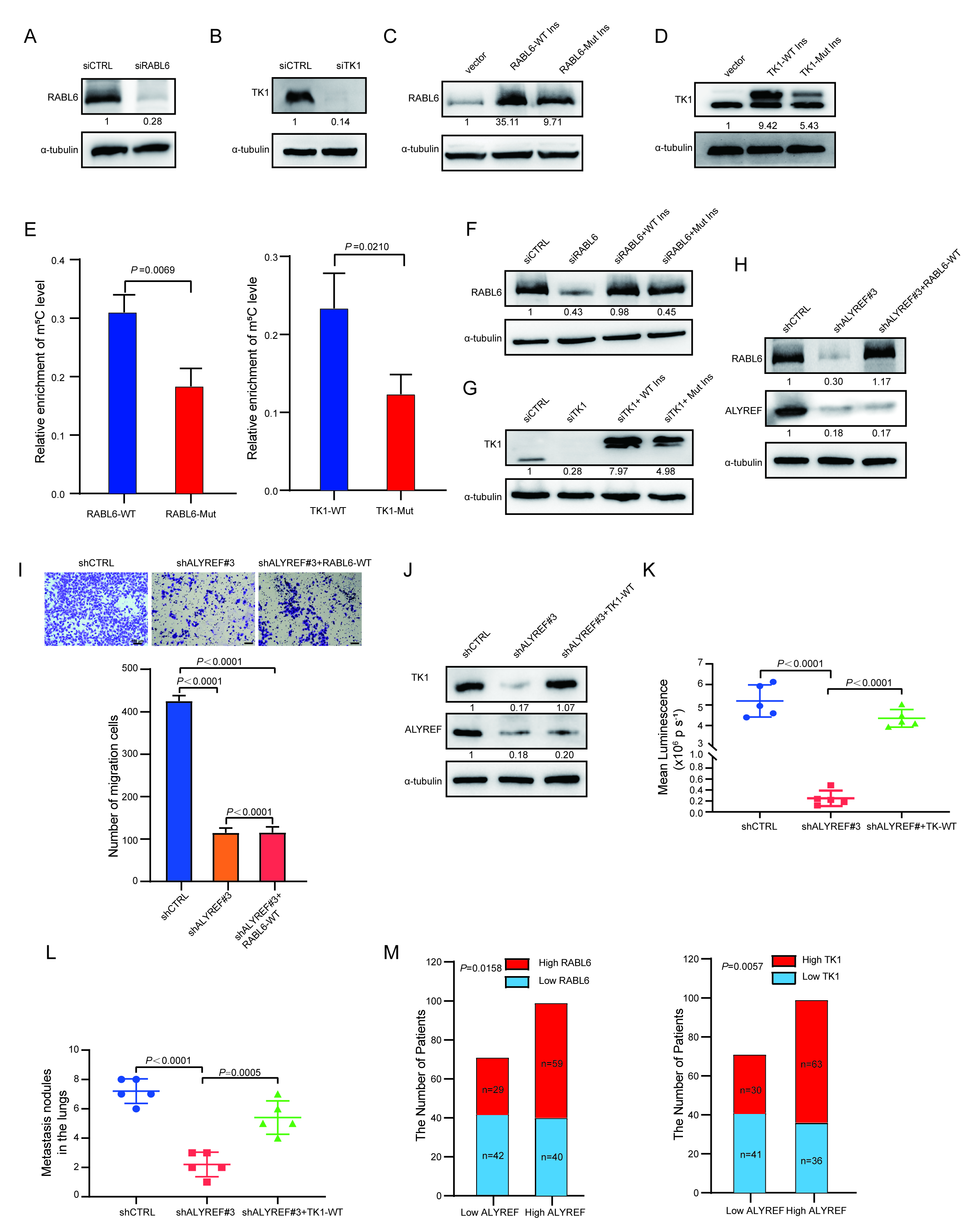

Supplement: Supplementary file 5 — Supplementary Figure 5 [file 41419_2023_5661_MOESM5_ESM.tif]

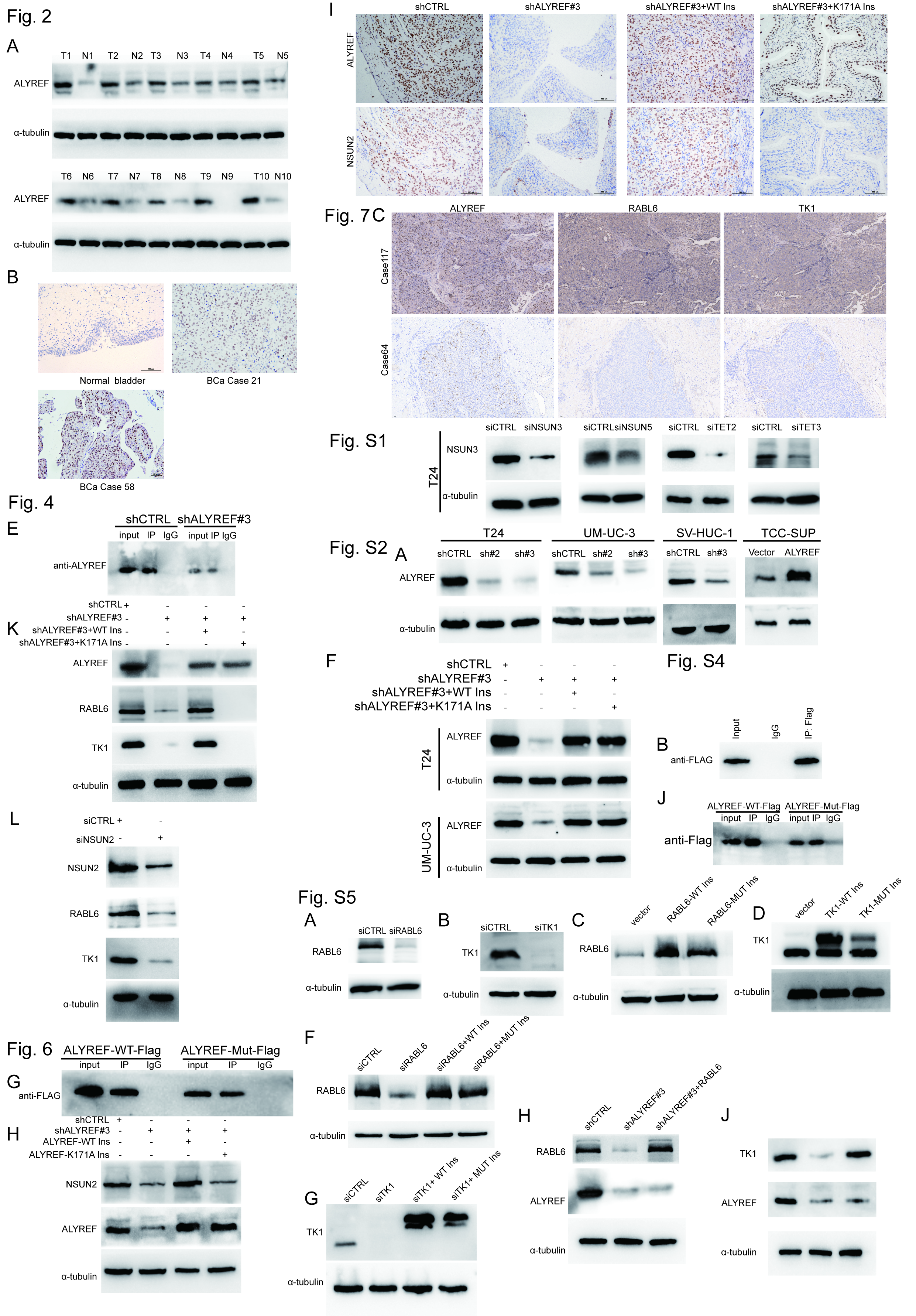

Supplement: Supplementary file 8 — Original Data File [file 41419_2023_5661_MOESM8_ESM.tif]
