# Supplementary material for: m5C-dependent cross-regulation between nuclear reader ALYREF and writer NSUN2 promotes urothelial bladder cancer malignancy through facilitating RABL6/TK1 mRNAs splicing and stabilization
Source: Cell Death Dis. 2023 Feb 18;14(2):139. doi: 10.1038/s41419-023-05661-y (PMC9938871; doi:10.1038/s41419-023-05661-y)
Supplement: Supplementary file 7 — The supplementary information [file 41419_2023_5661_MOESM7_ESM.docx]

**Supplementary Methods**

**Organoid model**

Fresh UCB tissue from a patient undergoing radical cystectomy at SYSUCC was stored in serum-free DMEM/F-12 (Invitrogen), which contains supplements, as shown by Suk Hyung Lee *et al*. [1] After cutting the sample into 1-2 mm small pieces and washing with sterile HBSS (Invitrogen), the UCB tissue was incubated with collagenases IV (Sigma) for half an hour. The suspension was then passed through a 100 μm cell strainer (Corning). After washing three times, the suspension was cultured on 24-well culture plates with matrigel (Corning) and incubated at 37 ℃. We explored the function of ALYREF through lentiviral infection like Deng *et al.* [2] To observe the effect of ALYREF on organoid formation, we calculated the ratio of the area of the organoid before and after infection. The ratio was compared between organoids treated with shCTRL and shALYREF#3.

**Colony-formation assay**

Five hundred cells were plated per well in 6-well plates. After cultured for 10-14 days, colonies were fixed and stained before counting.

**Migration assays**

In migration assays, 500µl of 10% FBS containing DMEM or RIPA 1640 medium was added to the lower chamber. A total of 5× 10^4^ cells suspended in 500 µl of serum-free medium were plated in a Boyden chamber (BD Biosciences, New Jersey, USA) in the insert of a 24-well plate. Twenty-four hours of incubation later, migration cells were fixed and stained before counting under a microscope.

**Invasion assay**

For invasion assay, a total of 5×10^4^ cells suspended in 500 µl of serum-free medium were plated in a Boyden chamber (BD Biosciences, New Jersey, USA) with matrigel (Corning) in the insert of a 24-well plate. 500µl of 10% FBS containing medium was added to the lower chamber. Incubated for twenty-four hours and the cells were fixed and stained before counting under a microscope.

**Animal models**

In different models, animals were randomly selected and at least five animals in each condition. The researches involving animals were blinded. All of the BALB/c nude mice (Four-week-old, female, 18–20 g) used in the tail-vein assays of lung metastasis, the subcutaneous and orthotopic xenograft models, were acquired from Vital River Laboratory (Beijing, China).

For the orthotopic xenograft model, the mice were anesthetized with isoflurane (Baxter Healthcare Corporation, Guayama, Puerto Rico, 00784) throughout the surgery protocol. A 24G closed IV catheter was inserted into the urethra of mice. After injection of 100 µl of hydrochloric acid (0.1 mol/L) for approximately 20 s, 100 µl of sodium hydroxide (0.1 mol/L) was infused and retained for the same time. One hundred microliters of PBS were used to wash the bladder before luciferase-transduced 1×10^6^ T24 cells were injected into the bladder, and then the urethra was ligated with sutures to maintain the cells for approximately 30 min. To image the tumors in the bladder, the mice were anaesthetized by isoflurane and injected with VivoGloluciferin solution (15 mg ml^−1^; Promega) 100µl intraperitoneally. Then, the mice of the orthotopic model were observed with the IVIS 200 imaging system. Six weeks later, the bladders were acquired from the mice.

For the subcutaneous xenograft model, 3×10^6^ T24 cells were injected into the right flank of each four-week-old BALB/c nude mouse. Tumor formation was evaluated after 4 weeks.

For the lung metastasis model, 1×10^6^ luciferase-transduced T24 cells were injected via the tail vein for each four-week-old Balb/c nude mouse. The mice were sacrificed to assess tumor growth 6 weeks later. To image the tumors in the bladder, the IVIS 200 imaging system was used to detect changes in the tumor after the mice were anesthetized by isoflurane and injected with 100 µl VivoGloluciferin solution (15 mg ml^−1^; Promega) intraperitoneally.

**Total RNA extraction and RNA-seq**

Total RNA of shCTRL and shALYREF#3 was extracted with TRIzol reagent (Invitrogen), and then the RNA samples were sent to RIBOBIO (GuangZhou, China) to conduct RNA-seq analysis. After quality control, RNA integrity evaluation by Agilent 2200 TapeStation (Agilent Technologies, USA) and purification by EpicentreRibo-Zero rRNA Removal Kit (illumina, USA), NEB Next Ultr RNA Library Prep Kit for Illumina (NEB, USA) was applied to generate mRNA sequencing libraries. The clean reads were mapped to the hg19 genome by HISAT2 with default parameters after dilution and purification. The aligned short reads were converted into read counts for each gene model by HTSeq. Differential expression was assessed by DEseq with |Fold change| cutoff = 1.5.

**Quantitative real-time polymerase chain reaction**

Total RNA of samples was extracted with TRIzol reagent (Invitrogen), and then 1 μg of RNA was reverse transcribed to cDNA according to the protocol of the PrimeScript RT reagent kit (TaKaRa). SYBR Green SuperMix (Roche, Basel, Switzerland) and an ABI Prism 7000 Sequence Detection System (Applied Biosystems) were used for qRT-PCR. Relative Quantification (ΔΔCT) method was used to analyze qRT-PCR data. Primer sequences are listed in Table S6.

**RNA immunoprecipitation**

The RIP process was conducted according to the protocol of the Magna RIP RNA-Binding Protein Immunoprecipitation Kit (Millipore). Cells were harvested and lysed in lysis buffer for half an hour at 4 °C. The lysates were collected after centrifugation and incubated overnight with 30µl of Protein-A/G agarose beads (Thermo-Fisher Scientific, Waltham, MA, USA) and antibodies. After extensive washing, the immunoprecipitated RNA was isolated by TRIzol reagent (Invitrogen) and used for qRT-PCR analysis, and the beads-bound proteins were isolated by boiling and western blotting. The primer sequences targeting m^5^C sites are listed in Table S6. The primaries antibodies for RIP used in our study were as follows: rabbit polyclonal anti-ALYREF (Cell Signaling Technology, Cat#: 12655; 1:200), rabbit polyclonal anti-Flag-HRP (Cell Signaling Technology, Cat#: 2368S; 1:200), anti-m^5^C antibody (Abcam, ab10805, 1:200).

**RNA m^5^C dot blot**

RNA was extracted from cells and then treated with deoxyribonuclease I (DNase). After concentration measurement, the RNA was loaded onto the Amersham Hybond N+ membrane (GE Healthcare) by a Bio-Dot apparatus (Bio-Rad, Hercules, CA, USA). After cross-linked by ultraviolet (5 min at 254 nm), the membrane was blocked by 5% milk and incubated at 4°C with anti-m^5^C antibody (Abcam, ab10805, 1:1000) overnight. followed by washed with TBST, the membrane was then incubated with secondary antibody. An enhanced chemiluminescence system (Tanon, Shanghai, China) was applied to show the signals on the membranes.

**Coimmunoprecipitation, silver staining, and** **mass spectrometry**

Protein from plasmid-transfected or wild type cells was harvested and rotated for 30 min at 4 ℃ with FLAG or ALYREF antibodies. Then, Sepharose-conjugated protein G magnetic beads (Thermo-Fisher Scientific, Waltham, MA, USA) were incubated with the samples for 24 h at 4 °C. After 4 washes, the beads were boiled and suspended in 1× SDS, and the proteins were collected. Western blotting, silver staining, or mass spectrometry was carried out for the subsequent analysis. Silver staining was conducted according to the protocol provided by the Fast Silver Stain Kit (Beyotime, Haimen, China). Mass spectrometry was completed by Wininnovate Bio (Shenzheng, China). The lyophilized peptide fractions were trapped and desalted by the Easy nLC 1200 system (ThermoFisher). The tandem mass spectrometry data were acquired through data-dependent acquisition mass spectrum techniques on a ThermoFisher Q Exactive mass spectrometer (ThermoFisher, USA). The primaries antibodies for IP used in our study were as follows: rabbit polyclonal anti-ALYREF (Cell Signaling Technology, Cat#: 12655; 1:200), rabbit polyclonal anti-Flag-HRP (Cell Signaling Technology, Cat#: 2368S; 1:200).

**Splicing analysis**

Forty-eight hours after transfected with plasmids, RNA of T24 cells was collected by TRIzol reagent (Invitrogen), and treated by DNAse Ⅰ (Promega). Reverse transcription was done with SuperScript^TM^ Ⅲ Reverse Transcriptase (ThermoFisher Scientific). RNAse H (ThermoFisher Scientific) was used to treat cDNA. SYBR Green SuperMix (Roche, Basel, Switzerland) was used for qRT-PCR. Unspliced pre-mRNA was detected by primers overlapping exon-intron boundaries, while spliced mature mRNA was measured by primers overlapping exon-exon boundaries (Table S6).

**Nuclear and cytoplasmic extraction**

Cytoplasmic and nuclear fractions were extracted following the protocol of the PARIS™ Kit (AM1556, Thermo Fisher Scientific, Waltham, USA). Briefly, the T24 cytoplasmic fraction was collected from the supernatant by lysis in cell fraction buffer and centrifugation at 500 × g for 3 min on ice. The nuclei were extracted, followed by washing of the pellet with cell fraction buffer. The nuclear and cytoplasmic RNA were then extraction and used for qRT-PCR.

**RNA stability assay**

shCTRL and shALYREF#3 cells were treated with actinomycin D (1 μg/mL, Sigma-Aldrich) and collected at different time (0, 2, 4, 6, 8h). After RNA extraction and reverse transcription, qRT-PCR was applied to measure mRNA remaining.

**Luciferase reporter assay**

When the density of shCTRL and shALYREF#3 T24 cells was 40% per well in 6-well plates, reporter plasmids containing wild-type or mutant 5’-UTR of RABL6 and reporter plasmids containing wild-type or mutant 3’-UTR of TK1 were transfected, respectively. When the density of wild-type T24 cells was 40% per well in 6-well plates, reporter plasmids containing wild-type or mutant 3’-UTR of NUSN2 were transfected. After 48-hour- incubation, cells were collected, and RNA was extracted and measured. A dual-luciferase reporter assay system (Promega, Madison, WI) was used to assess the effect of different reporter plasmids. Renilla Luciferase was used to normalized the relative luciferase activity. In ALYREF knockdown group, the relative Luc/Rluc ratio was further normalized to that of control sample.

Double immunofluorescence staining assay

The bladder cancer tissues were formalin-fixed and paraffin-embedded. Then, 4-µm thick tissue sections were cut for double immunofluorescence staining. After dewaxing and rehydration treatment, slides were incubated in Ethylenediaminetetraacetic Acid (EDTA) buffer (pH 8.0) and then boiled in a pressure cooker for 5 minutes. Primary antibodies for ALYREF and RABL6 or ALYREF and TK1 were added for incubation overnight at 4 ℃. After incubation with secondary antibody for 30 min at 37 ℃, slides were counterstained with DAPI (C1005, Beyotime, Shanghai, China) for 10 min in the dark at room temperature. After washed three times with PBS, OLYMPUS FV1000 confocal microscopy were used to observe results and capture the pictures. The primaries antibodies for double immunofluorescence staining used in our study were as follows: rabbit polyclonal anti-ALYREF (Cell Signaling Technology, Cat#: 12655; 1:200), mouse monoclonal anti-TK1 (Proteintech, Cat#: 67787-1-Ig; 1:250), mouse polyclonal anti-RABL6 (Abnova, Cat#: H00055684-A01; 1:200).

**Plasmid construction and transfection**

The ALYREF overexpression plasmid and shALYREF-insensitive WT and K171A mutant plasmids were obtained from GeneCopeia. The siRNA-insensitive WT or mutant plasmids of TK1 and RABL6 were purchased from GeneCreate (Wuhan, China). The 3’UTR of TK1 with a WT or mutant m^5^C site (chr17: 76170268) and the 5’UTR of RABL6 with a WT or mutant m^5^C site (chr9: 139702478) were integrated into the siRNA-insensitive WT plasmids of TK1 and RABL6, respectively. The pmirGLO–NSUN2-WT plasmid was constructed by inserting NSUN2 downstream of the pmirGLO vector. Cytosine (the methylated site) was mutated in pmirGLO–NSUN2-WT to generate the pmirGLO–NSUN2-mutant plasmid. Lipofectamine 3000 (Invitrogen, CA, USA) was used for plasmid transfection. A Lenti-Pac HIV packaging kit (GeneCopoeia, MD, USA) was used for lentivirus production and infection. To construct stable cell lines of T24, UM-UC-3, TCC-SUP and SV-HUC-1, lentivirus expressing shRNAs and genes were transfected into cells. Puromycin or neomycin was used for stable cells selection after several days of infection.

**Sequencing data applied from our previous study**

We applied transcriptome-wide RNA-BisSeq and RNA-Seq data of 36 UCBs and in 29 adjacent normal tissues from SYSUCC^2^. The criteria of m^5^C sites: coverage ≥10 in at least 30 samples, including ≥10 normal, ≥10 tumour and ≥5 paired cancer and non-cancerous samples. Differential m^5^C sites were defined with the following criteria: *P*<0.05 (two-sided unpaired Wilcoxon and Mann–Whitney tests), mean m^5^C level difference≥0.05 (tumor and normal samples, paired cancer and non-cancerous samples) and a uniform tendency of m^5^C level change in ≥5 paired samples. Transcriptome-wide RNA-BisSeq data of T24 cells in siCTRL and siNSUN2 from Chen *et al.* [3] were also applied. mRNAs regulated by m^5^C were defined as mean m5C level difference > 0.1, *P* value < 0.05.

**References**

1. Lee SH, Hu W, Matulay JT, Silva MV, Owczarek TB, Kim K, et al. Tumor Evolution and Drug Response in Patient-Derived Organoid Models of Bladder Cancer. Cell. 2018; 173: 515-28.
2. Deng MH, Wang N, Li ZY, Chen RX, Duan JL, Peng YL, et al. FXR1 can bind with the CFIm25/CFIm68 complex and promote the progression of urothelial carcinoma of the bladder by stabilizing TRAF1 mRNA. Cell Death Dis. 2022; 13: 170.
3. Chen X, Li A, Sun BF, Yang Y, Han YN, Yuan X, et al. 5-methylcytosine promotes pathogenesis of bladder cancer through stabilizing mRNAs. Nat Cell Biol. 2019; 21: 978-90.

**Table S1.** Clinical characteristics of UCB^a^ samples for western blotting

| Patient No. | Age at diagnosis | Gender | Pathological T-stage | Pathological N-stage |
| --- | --- | --- | --- | --- |
| 1 | 71 | male | 2 | 0 |
| 2 | 47 | male | 2 | 0 |
| 3 | 85 | Male | 2 | 0 |
| 4 | 76 | male | 2 | 0 |
| 5 | 52 | Male | 4 | 3 |
| 6 | 64 | Male | 3 | 3 |
| 7 | 62 | Female | 1 | 0 |
| 8 | 49 | Male | 3 | 1 |
| 9 | 56 | Male | 1 | 0 |
| 10 | 67 | male | 1 | 0 |

a: urothelial carcinoma of the bladder.

**Table S3.** Clinical characteristics of UCB^a^ samples for organoid model

| Patient No. | Age at diagnosis | Gender | Pathological T-stage | Pathological N-stage |
| --- | --- | --- | --- | --- |
| 1 | 57 | male | 1 | 0 |

a: urothelial carcinoma of the bladder.

**Table S4.** Clinical characteristics of UCB^a^ samples for m^5^C-RIP-PCR

| Patient No. | Age at diagnosis | Gender | Pathological T-stage | Pathological N-stage |
| --- | --- | --- | --- | --- |
| 1 | 58 | male | 2 | 0 |
| 2 | 62 | male | 2 | 0 |
| 3 | 59 | female | 3 | 0 |
| 4 | 64 | male | 2 | 0 |
| 5 | 62 | male | 3 | 0 |

a: urothelial carcinoma of the bladder.

**Table S5.** Sequences of siRNA and shRNA

| Name | Primer sequence (5'-3') |
| --- | --- |
| siNSUN2 | CACGTGTTCACTAAACCCTAT |
| siRABL6 | GGCCTAAAGTACCTTCATA |
| siTK1 | ACAAGTGCCTGGTGATCAA |
| ALYREF-shRNA2 | GCTTGTCACGTCACAGATTGA |
| ALYREF-shRNA3  siNSUN3  siNSUN5  siTET2  siTET3 | GCGTAAACAGAGGTGGCATGA  CUCUGGGUCUGUUUGGAAUTT  GACCTGCTCCGATGATGTA  GUACAGAAUAUAAAUCGUA  CGCCCTTGAGCTCCAACGAGAA |

**Table S6.** Primers for PCR

| Primer Name | Direction | Primer sequence (5'-3') |
| --- | --- | --- |
| RIP-NSUN2 | Forward | TGCTTTACAGGCCACAGGCT |
|  | Reverse | ACCCCTCACACGCAAAACT |
| RIP-TK1 | Forward | GGCTCAAAGCCCTTCCTACC |
|  | Reverse | TAAGCTACAGCAGAGGCGTG |
| RIP-RABL6 | Forward | TGACTCCTGGAGAGCGGT |
|  | Reverse | GGGTGCGACTGGCACCATGT |
| GAPDH | Forward | TGCACCACCAACTGCTTAGC |
|  | Reverse | GGCATGGACTGTGGTCATGAG |
| U6 | Forward | CAGCACATATACTAAAATTGGAACG |
|  | Reverse | ACGAATTTGCGTGTCATCC |
| ALYREF | Forward | CTGTTCCTAAGCTGCGACCA |
|  | Reverse | GGCCAAAACAACTTCCCGAC |
| NSUN2 | Forward | GGTATCCTGAAGAACTTGCC |
|  | Reverse | ATCTTATGATGAGGCCGCA |
| TK1 | Forward | TGTAGCGAGTGTCTTTGGCATA |
|  | Reverse | GGGCAGATCCAGGTGATTCTC |
| RABL6 | Forward | GCAACAGCGTCTCCCTCTGA |
|  | Reverse | AGGTTCCTCCTACTTCCGCT |
| PSIP1 | Forward | CAACAGGCAGCAACTAAACAATC |
|  | Reverse | TCATGGTCGGTATCTTCCTTTGA |
| MARCKS | Forward | CCAGTTCTCCAAGACCGCAG |
|  | Reverse | TCTCCTGTCCGTTCGCTTTG |
| CCNG1 | Forward | GAGTCTGCACACGATAATGGC |
|  | Reverse | GTGCTTGGGCTGTACCTTCA |
| CYP51A1 | Forward | GAAACGCAGACAGTCTCAAGA |
|  | Reverse | ACGCCCATCCTTGTATGTAGC |
| MSMO1 | Forward | GCAAGATGCTTTGGTTGTGC |
|  | Reverse | AATGGTCACCCATGCCCAAA |
| COL4A1 | Forward | GGGATGCTGTTGAAAGGTGAA |
|  | Reverse | GGTGGTCCGGTAAATCCTGG |
| PEG10 | Forward | GAGCACCAGGGATTTCTCAGT |
|  | Reverse | GGTAGTTGTGCATCAGGTAGTG |
| PADI2 | Forward | ACCTCTGGACCGATGTCTACA |
|  | Reverse | TCCCTTCCTCGTCATAGTAGTTG |
| RTN3 | Forward | ATGACGGACTTGTAGATCCTGA |
|  | Reverse | ACTGGGTTTGTCTTTGGCAC |
| exon-intron RABL6 | Forward | GCCAAGGGGGTGCAGTACAA |
|  | Reverse | AAGCAGGTGAGACCGACACC |
| exon-exon RABL6 | Forward | GGGAAGATGTTTTCCGCCCT |
|  | Reverse | CCTGTCTCCCCGGATCACTA |
| exon-intron TK1 | Forward | AGCTGCATTAACCTGCCCAC |
|  | Reverse | ATGCCTGGACACAGGCTATC |
| exon-exon TK1 | Forward | GTCATAGGCATCGACGAGGG |
|  | Reverse | CCAGTGCAGCCACAATTACG |
| RBM26 | Forward | GGAAGTACACACTTAAAATATCAGACTGG |
|  | Reverse | GCTAACATTTGGTGAATAAGAAAACATC |
| SLC39A9 | Forward | GGAGCCTTGAACTCCGGCA |
|  | Reverse | AGTATGGCTATTTCTGTCTGCTGA |
| NUMB | Forward | GTGATGCTGGCTGGGAGATGG |
|  | Reverse | GACCGAGTGGGGTCAATCTTCT |

**Table S7**. m^5^C sites of *NSUN2*, *RABL6* and *TK1* from Chen *et al.*, Huang *et al.* and Yang *et al.* studies.

|  | | | Chen *et al.* | | | | Huang *et al.* | | | | | | | Yang *et al.* | | | | |
| --- | --- | --- | --- | --- | --- | --- | --- | --- | --- | --- | --- | --- | --- | --- | --- | --- | --- | --- |
|  |  |  | T24 Control | | T24 siNSUN2 | | HeLa Control | | | HeLa  NSUN2 knockout | | HeLa  NSUN2 rescue | | HeLa Control | | | HeLa siNSUN2 | |
| Chromosome | Position | Gene name | Coverage | m^5^C level | Coverage | m^5^C level | Coverage | m^5^C level | P-value | Coverage | m^5^C level | Coverage | m^5^C level | Coverage | m^5^C level | P-value | Coverage | m^5^C level |
| 9 | 139702478 | *RABL6* | 4847 | 0.353 | 3607 | 0.139 | 295 | 0.66 | 0.00E+00 | 265 | 0.00 | 55 | 0.27 | 1890 | 0.66 | 0 | 2762 | 0.33 |
| 17 | 76170268 | *TK1* | 1523 | 0.164 | None | 0 | 290 | 0.12 | 1.40E-71 | 231 | 0.00 | 130 | 0.07 | 627 | 0.19 | 2.18E-241 | 646 | 0.07 |
| 5 | 6600023 | *NSUN2* | 2940 | 0.213 | None | 0 | 600 | 0.27 | 0.00E+00 | 595 | 0.00 | 179 | 0.12 | 5518 | 0.46 | 0 | 819 | 0.26 |

**Table S8.** m^5^C methylated *NSUN2*, *RABL6* and *TK1* in ALYREF-RIP- bisulfite-sequencing.

|  |  |  |  | ALYREF-RIP-BS | | |
| --- | --- | --- | --- | --- | --- | --- |
| Position | Strand | Gene Symbol | Gene Type | State_rep1 | State_rep2 | Overall m^5^C Level |
| 6600023 | - | *NSUN2* | mRNA | 1 | 1 | 0.357576 |
| 139702478 | + | *RABL6* | mRNA | 1 | 1 | 0.591837 |
| 76170268 | - | *TK1* | mRNA | 1 | 1 | 0.318841 |

**Table S9.** RIP Peaks of ALYREF-Flag-RIP seq in *NSUN2*, *RABL6* and *TK1* in Yang *et al.*

| Gene name | m^5^C Position | ALYREF-RIP-rep_peaks | Chromosome | Start | End | -log10(p value) | Fold Enrichment |
| --- | --- | --- | --- | --- | --- | --- | --- |
| *NSUN2* | 6600023 | rep1 | chr5 | 6599459 | 6600369 | 64.78745 | 11.91136 |
|  |  | rep2 | chr5 | 6599452 | 6600367 | 78.70029 | 11.06719 |
| *RABL6* | 139702478 | rep1 | chr9 | 139702376 | 139702908 | 233.5534 | 18.58974 |
|  |  | rep2 | chr9 | 139702374 | 139702914 | 308.5561 | 18.84532 |
| *TK1* | 76170268 | rep1 | chr17 | 76170288 | 76171330 | 206.7656 | 11.67969 |
|  |  | rep2 | chr17 | 76170283 | 76171330 | 217.4195 | 10.63694 |

**Table S10.** The upstream and downstream 25 bp sequences of the m^5^C sites in *RABL6*, *TK1* and *NSUN2*.

| Gene | Sequences of the upstream and downstream 25 bp of the m^5^C sites |
| --- | --- |
| *RABL6* | GAGCGGAGCAGCCGCGGCTGAGGTTCCCGAGTCGCCGCTCGGGGCTGCGCT |
| *TK1* | ACCAAGATGGGTGGCACCAACCTTGCTGGGACTTGGATCCCAGGGGCTTAT |
| *NSUN2* | TTTGCGTGTGAGGGGTGTGGGCCCCCGCTGCCTTGGGCCTGCTCACCGGGG |

**Table S11**. ALYREF interacted proteins identified by mass spectrum from Khan *et al.* and in T24 cells.

| Representative splicing factors | Khan *et al.*  ALYREF IP | T24 cells  Flag IP | T24 cells  ALYREF IP | TREX complex components | Khan *et al.* | T24 cells  Flag IP | T24 cells  ALYREF IP |
| --- | --- | --- | --- | --- | --- | --- | --- |
| Symbol | Peptides | Peptides | Peptides | Symbol | Peptides | Peptides | Peptides |
| SFPQ | 8 | 6 | 124 | Uap56 | 49 | 3 | 16 |
| PUF60 | 8 | 17 | 5 | THOC1 | NA | 11 | NA |
| SRSF1 | 5 | 1 | 35 | THOC2 | NA | 32 | 11 |
| SF3A1 | 10 | 10 | NA | THOC5 | 78.5 | 2 | NA |
| SF3B1 | 9 | 22 | 24 | THOC6 | NA | NA | 9 |
| SRSF3 | NA | NA | 24 |  |  |  |  |

**Supplementary Figure Legends**

**Fig. S1 m^5^C regulators are predominantly upregulated in UCB.** **A** The expression levels of m^5^C regulators (fold change >1.1, *P* value < 0.05, occurrence rate> 50%) in 22 paired UCB tumor tissues and adjacent non-neoplastic tissues of SYSUCC cohort. Data represent the mean ± S.D., and a two-tailed paired Student’s *t* test was applied to determine the *P* value. **B** The expression levels of m^5^C regulators (fold change >1.1, *P* value < 0.05, occurrence rate> 50%) in 19 paired UCB tumor tissues and adjacent normal tissues of the TCGA cohort. Data represent the mean ± S.D. A two-tailed paired Student’s *t* test was applied to determine the *P* value. **C** Western blotting indicating the effective knockdown of NSUN3, NSUN5, TET2 and TET3 by siRNAs in T24 cells. The expression was normalized by α-tubulin expression. **D** Migration assays indicating migration abilities after the knockdown of NSUN3, NSUN5, TET2 and TET3. Left: representative images; Right: statistical charts of migration cell numbers. Scale bars, 100 μm. Data represent the mean ± S.D., n=3, and a two-tailed unpaired Student’s *t* test was applied to determine the *P* value. **E** Colony- formation assays indicating cell growth ability after the knockdown of NSUN3, NSUN5, TET2 and TET3. Left: representative images; Right: histograms of colony numbers. Data represent the mean ± S.D., n=3, and a two-tailed unpaired Student’s *t* test was applied to determine the *P* value.

**Fig. S2 ALYREF enhances UCB cell proliferation and invasion *in vitro*.** **A** Western blotting indicating the effective knockdown of ALYREF by shALYREF#2 and shALYREF#3 in T24, UM-UC-3 and SV-HUC-1 cells, and the efficient overexpression in TCC-SUP cells. The expression was normalized by α-tubulin expression. **B** Colony- formation assays indicating cell growth ability after the knockdown of ALYREF. Top: representative images; Bottom: histograms of colony numbers. Data represent the mean ± S.D., n=3, and a two-tailed unpaired Student’s *t* test was applied to determine the *P* value. **C** Migration assays indicating migration abilities after the knockdown of ALYREF. Top: representative images; Bottom: statistical charts of migration cell numbers. Scale bars, 100 μm. Data represent the mean ± S.D., n=3, and a two-tailed unpaired Student’s *t* test was applied to determine the *P* value. **D** Colony- formation assays indicating cell growth ability after the knockdown of ALYREF in SV-HUC-1 cells. Left: representative images; Right: histograms of colony numbers. Data represent the mean ± S.D., n=3, and a two-tailed unpaired Student’s *t* test was applied to determine the *P* value. **E** Migration assays indicating migration abilities after the knockdown of ALYREF in SV-HUC-1 cells. Left: representative images, Scale bars, 100 μm; Right: histograms of migration cell numbers. Data represent the mean ± S.D., n=3, and a two-tailed unpaired Student’s *t* test was applied to determine the *P* value. **F** Western blotting assays showing the effect of ALYREF with a WT m^5^C site on the restoration of protein expression in ALYREF-knockdown cells relative to ALYREF with K171A mutant and were normalized by α-tubulin expression. **G** CCK-8 assay showing the effect of ALYREF with a WT m^5^C site on the restoration of growth rate in ALYREF-knockdown cells relative to ALYREF with K171A mutant in T24 (Left) and UM-UC-3 (Right) cells. Data represent the mean ± S.D., n=3. A two-tailed unpaired Student’s *t* test was applied to calculate the *P* value. **H** Invasion assay showing the effect of ALYREF with a WT m^5^C site on the restoration of cell invasiveness in ALYREF-knockdown cells relative to ALYREF with K171A mutant. Left: representative images of invasive cells in T24 (Top) and UM-UC-3 (Bottom) cells; Scale bars, 100 μm. Right: histograms of the number of invasive cells. Data represent the mean ± S.D., n=3. A two-tailed unpaired Student’s t test was applied to calculate the *P* value. **I** Colony formation assays showing the colony formation ability transfected with vector and overexpression of ALYREF in TCC-SUP cells. Data represent the mean ± S.D., n=3. A two-tailed unpaired Student’s *t* test was applied to determine the *P* value. **J** Migration assays showing the cell migration ability transfected with vector and overexpression of ALYREF in TCC-SUP cells. Scale bars, 100 μm. Data represent the mean ± S.D., n=3. A two-tailed unpaired Student’s *t* test was applied to determine the *P* value.

**Fig. S3. ALYREF promotes UCB pathogenesis and metastasis potential as an m^5^C reader *in vivo*.** **A** Statistical results for the orthotopic bladder weight in different groups. Data show the mean ± S.D. The *P* values were calculated by a two-tailed unpaired Student’s *t* test. n=5, ns: no significance. **B** Hematoxylin-eosin staining of orthotopic bladder tumors in different groups. Scale bars, 1.25 mm. **C** Statistical results for the mean tumor weight in different groups of the subcutaneous xenograft model. Data indicates the mean ± S.D. The *P* values were calculated by a two-tailed unpaired Student’s *t* test. n=6, ns: no significance.

**Fig. S4. Potential mRNAs regulated by ALYREF and proteins bound to ALYREF.**

**A** Functional enrichment analysis of KEGG showing the genes related to ALYREF knockdown enriched in canonical cancer-related pathways. **B** RIP assays showing ALYREF interacted with the m^5^C sites of *RABL6* and *TK1* mRNA. Fold enrichment showed *RABL6* or *TK1* mRNA levels associated with ALYREF compared to an input control. IgG antibody used as a control. Data show the mean ± S.D., n=3. The *P* values were calculated by a two-tailed unpaired Student’s *t* test. **C** Silver staining assays showing the protein bands binding to exogenous ALYREF. **D** Functional enrichment analysis of KEGG from mass spectrometry analysis showing several spliceosome factors precipitated by ALYREF. **E** Integrative- genomics-viewer tracks representing the intron reads regions of *RABL6* (Left)and *TK1* (Right) in shCTRL and shALYREF#3 RNA-seq data. **F** Splicing efficiency of *RABL6* and *TK1* was reduced in mature mRNA when ALYREF knockdown. Left: qRT-PCR showing the splicing efficiency of *RABL6* and *TK1* in mature mRNA when ALYREF knockdown. Right: qRT-PCR showing the splicing efficiency of *RABL6* and *TK1* in premature mRNA when ALYREF knockdown. Schematic illustration showing the qRT-PCR primers designed across exon-intron junction and across exon-exon junction. Data show the mean ± S.D., n=3. The *P* values were calculated by a two-tailed unpaired Student’s *t* test. **G** Splicing efficiency of *RABL6* and *TK1* was reduced in mature mRNA when NSUN2 knockdown. Left: qRT-PCR showing the splicing efficiency of *RABL6* and *TK1* in mature mRNA. Right: qRT-PCR showing the splicing efficiency of *RABL6* and *TK1* in pre-mRNA. Schematic illustration showing the qRT-PCR primers designed across exon-intron junction and across exon-exon junction. Data show the mean ± S.D., n=3. The *P* values were calculated by a two-tailed unpaired Student’s *t* test. **H** Luciferase reporter assay showing the relative luciferase mRNA (Left) and activity (Right) level of luciferase reporter gene with WT-m^5^C-site RABL6 (RABL6-WT) or MUT-m^5^C-site RABL6 (RABL6-MUT). Data represent the mean ± S.D., n=3. A two-tailed unpaired Student’s *t* test was applied to determine the *P* value. **I** Luciferase reporter assay showing the relative luciferase mRNA (Left) and activity (Right) level of luciferase reporter gene with WT-m^5^C-site TK1 (TK1-WT) or MUT-m^5^C-site TK1 (TK1-MUT). Data represent the mean ± S.D., n=3. A two-tailed unpaired Student’s *t* test was applied to determine the *P* value. **J** ALYREF-Flag-RIP assays showing that ALYREF K171A mutant did not affected the binding ability to RNAs in general. Upper panel: Western blotting shows Flag IP efficiency between ALYREF WT and K171A mutant. Bottom panel: Relative enrichment representing *RABL6*, *RBM26*, *SLC39A9* or *NUMB* mRNA levels associated with ALYREF compared to an input control. IgG antibody used as a control. Data show the mean ± S.D., n=3. The *P* values were calculated by a two-tailed unpaired Student’s *t* test.

**Fig. S5 Hypermethylated *RABL6* and *TK1* promote UCB pathogenesis. A** Western blotting showing the knockdown efficiency of RABL6 in siCTRL and siRABL6. The expression was normalized by α-tubulin expression. **B** Western blotting showing the knockdown efficiency of TK1 in siCTRL and siTK1. The expression was normalized by α-tubulin expression. **C** Western blotting indicating the expression level of RABL6, siRABL6-insensitive wild-type RABL6 (WT Ins) and its m^5^C site defective mutant (Mut-Ins) in T24 cells. The expression was normalized by α-tubulin expression. **D** Western blotting indicating the expression level of TK1, siTK1-insensitive wild-type TK1 (WT Ins) and its m^5^C site defective mutant (Mut-Ins) in T24 cells. The expression was normalized by α-tubulin expression. **E** m^5^C-RIP-qRT-PCR showing the relative enrichment of m^5^C level in *RABL6* or *TK1* mRNA with a WT or mutant m^5^C-site. Left: m^5^C-RIP-qRT-PCR showing the relative enrichment of m^5^C level in wild type *RABL6* containing m^5^C-site compared with m^5^C-site mutant *RABL6*. Right: The relative enrichment of m^5^C level in wild type *TK1* containing m^5^C-site compared with m^5^C-site mutant *TK1*. Data represent the mean ± S.D., n=3, and a two-tailed unpaired Student’s *t* test was applied to determine the *P* value. **F** Western blotting indicating the expression level of RABL6 in siCTRL and siRABL6 T24 cells, which expressing siRABL6-insensitive WT RABL6 (WT Ins) and the m^5^C site defective mutant (Mut-Ins). The expression was normalized by α-tubulin expression. **G** Western blotting indicating the expression level of TK1 in siCTRL and siTK1 T24 cells, which expressing siTK1-insensitive WT TK1 (WT Ins) and the m^5^C site defective mutant (Mut-Ins). The expression was normalized by α-tubulin expression. **H** Western blotting assays indicating the expression level of RABL6 and ALYREF in shCTRL and shALYREF#3 T24 cells, which expressing RABL6 with WT m^5^C-site. The expression was normalized by α-tubulin expression. **I** Migration assay showing the effect of RABL6 with a WT m^5^C site on the restoration of cell migration in ALYREF-knockdown cells. Upper panel: representative images of migration cells; Bottom panel: histograms of migration cells. Data show the mean ± S.D., n=3. The *P* values were calculated by a two-tailed unpaired Student’s *t* test. **J** Western blotting assays indicating the expression level of TK1 and ALYREF in shCTRL and shALYREF#3 T24 cells, which expressing TK1. The expression was normalized by α-tubulin expression. **K** statistical results for the mean bioluminescence signals in different groups at the 6th week. Data show the mean ± S.D. The *P* values were calculated by a two-tailed unpaired Student’s *t* test. n=5. **L** Statistical results for the number of metastatic nodules in the lung among different groups at the 6th week. Data show the mean ± S.D, The *P* values were calculated by a two-tailed unpaired Student’s *t* test. n= 5. **M** The correlation between the expression levels of ALYREF and regulated genes, including RABL6 (Left) and TK1 (Right), in 170 cases of UCB from SYSUCC. Data represent the mean ± S.D. The *P* values were determined by a two-tailed unpaired Student’s *t* test.
